# A high-throughput RNA-Seq approach to elucidate the transcriptional response of *Piriformospora indica* to high salt stress

**DOI:** 10.1038/s41598-021-82136-0

**Published:** 2021-02-18

**Authors:** Nivedita, Abdul Rawoof, Nirala Ramchiary, Malik Z. Abdin

**Affiliations:** 1 Department of Biotechnology, Jamia Hamdard, New Delhi, India; 2 School of Life Sciences, Jawaharlal Nehru University, New Delhi, India

**Keywords:** Fungi, Fungal genomics, Biotechnology, Microbiology, Molecular biology

## Abstract

*Piriformospora indica*, a root endophytic fungus, augments plant nutrition and productivity as well as protects plants against pathogens and abiotic stresses. High salinity is a major problem faced by plants as well as by microbes. Until now, the precise mechanism of salt stress tolerance in *P. indica* has remained elusive. In this study, the transcriptomes of control and salt-treated (0.5 M NaCl) *P. indica* were sequenced via the RNA-seq approach. A total of 30,567 transcripts and 15,410 unigenes for *P. indica* were obtained from 7.3 Gb clean reads. Overall 661 differentially expressed genes (DEGs) between control and treated samples were retrieved. Gene ontology (GO) and EuKaryotic Orthologous Groups (KOG) enrichments revealed that DEGs were specifically involved in metabolic and molecular processes, such as “response to salt stress”, “oxidoreductase activity”, “ADP binding”, “translation, ribosomal structure and biogenesis”, “cytoskeleton”, and others. The unigenes involved in “cell wall integrity”, “sterol biosynthesis”, and “oxidative stress” such as Rho-type GTPase, hydroxymethylglutaryl-CoA synthase, and thioredoxin peroxidase were up-regulated in *P. indica* subjected to salt stress. The salt-responsive DEGs have shown that they might have a potential role in salt stress regulation. Our study on the salt-responsive DEGs established a foundation for the elucidation of molecular mechanisms related to *P. indica* stress adaptation and a future reference for comparative functional genomics studies of biotechnologically important fungal species.

## Introduction

Eukaryotic organisms are often exposed to extreme conditions in their natural environment such as pH changes, osmotic stress, thermal stress, free oxide radicals, and toxic chemicals. Soil salinization is one of the most detrimental factors that cause osmotic stress and oxidative damage in various species^1–3^. Approximately 77 million hectares (5%) of cultivated lands worldwide are affected by salinity which results in a huge loss of agricultural production^4^. High salinity results in impaired metabolic and physiological activities in plants due to the reduction in osmotic potential, ion toxicity, and nutrient imbalance which cause damage to the cell organelles and disruption of photosynthesis, respiration, and protein synthesis^3,5^. Therefore, it is critically important to improve the stress tolerance and defense mechanism of plants for sustainable crop production in salinity affected regions.

The high saline environment also negatively affects the microorganisms; however, some microbes are equipped with special strategies to endure these stresses^1,6^. Various fungal species have been reported to exhibit halotolerance and their unique adaptability to the saline environment^7,8^. The detailed study of the mechanism of adaptation in these halotolerant species might help in the development of crop plants with higher salt tolerance^9^. Endophytic mycorrhizal fungi develop mutually beneficial relationships with a wide range of terrestrial plants and have been exploited to alleviate salt stress in crops^10^. It is observed that the heterologous expression of specific genes isolated from halotolerants e.g. *Debaryomyces hansenii,* and *Aspergillus glaucus*, significantly improved the salt tolerance of *Saccharomyces cerevisiae, Arabidopsis,* and tobacco^11,12^.

*Piriformospora indica* is an axenically cultivable phytopromotional, biotrophic endosymbiont that belongs to order Sebacinales (Basidiomycota)^13^. This root endophyte can colonize a variety of higher plants and benefits host plants with growth-promotion, disease resistance as well as stress tolerance^14^. Extensive studies have provided evidence that *P. indica* improves crop tolerance against various abiotic stresses such as salinity, drought, heat, cold, and heavy metal toxicity^15–19^. For instance, a cyclophilin like A protein of *P. indica* (PiCyPA) and PiHOG1 have shown a significant response to various abiotic stresses including salinity^20,21^. The alleviation of salt stress in plants by *P. indica* is well documented^22–24^. Apart from that, *P. indica* colonization activates defense-related genes (*PR, LOX2,* and *ERF1*), abiotic stress-responsive genes (*DREB2A, CBL1,* and *RD29A*), and osmoprotectants (e.g. proline, glycinebetaine) in host plants^17,19,25,26^.

High-throughput next-generation sequencing (NGS) technologies, with higher efficiency, enormous data production, and affordable cost, offer a better understanding of the mechanisms underlying the molecular diversity of non-model organisms^27^. The shorter reads with greater coverage generated through NGS technologies could facilitate better knowledge of the rich transcriptomes of various fungal species^28,29^.

Genomic information related to *P. indica* has been revealed with the release of genome sequence of the fungus^30^. However, there is rather limited information available on *P. indica* exposed to environmental stresses at the genomic and transcriptomic level. The objectives of the present study were to obtain a deeper insight into the potential ability of the fungus to combat salt stress via profiling of differentially expressed transcript/genes in *P. indica* during salt stress and to provide valuable genetic resources to aid further research at the molecular and genetic level. We performed RNA sequencing and de novo assembly of the transcripts and compared the generated transcriptome profile of *P. indica* grown with and without salt stress. We have identified a number of salt-responsive DEGs and performed their functional annotation. The present transcriptome profile would contribute to further enrich the power of comparative genomics information in the basidiomycetes group.

## Results

### Effect of salt treatment on *P. indica* biomass

**Figure 1:**
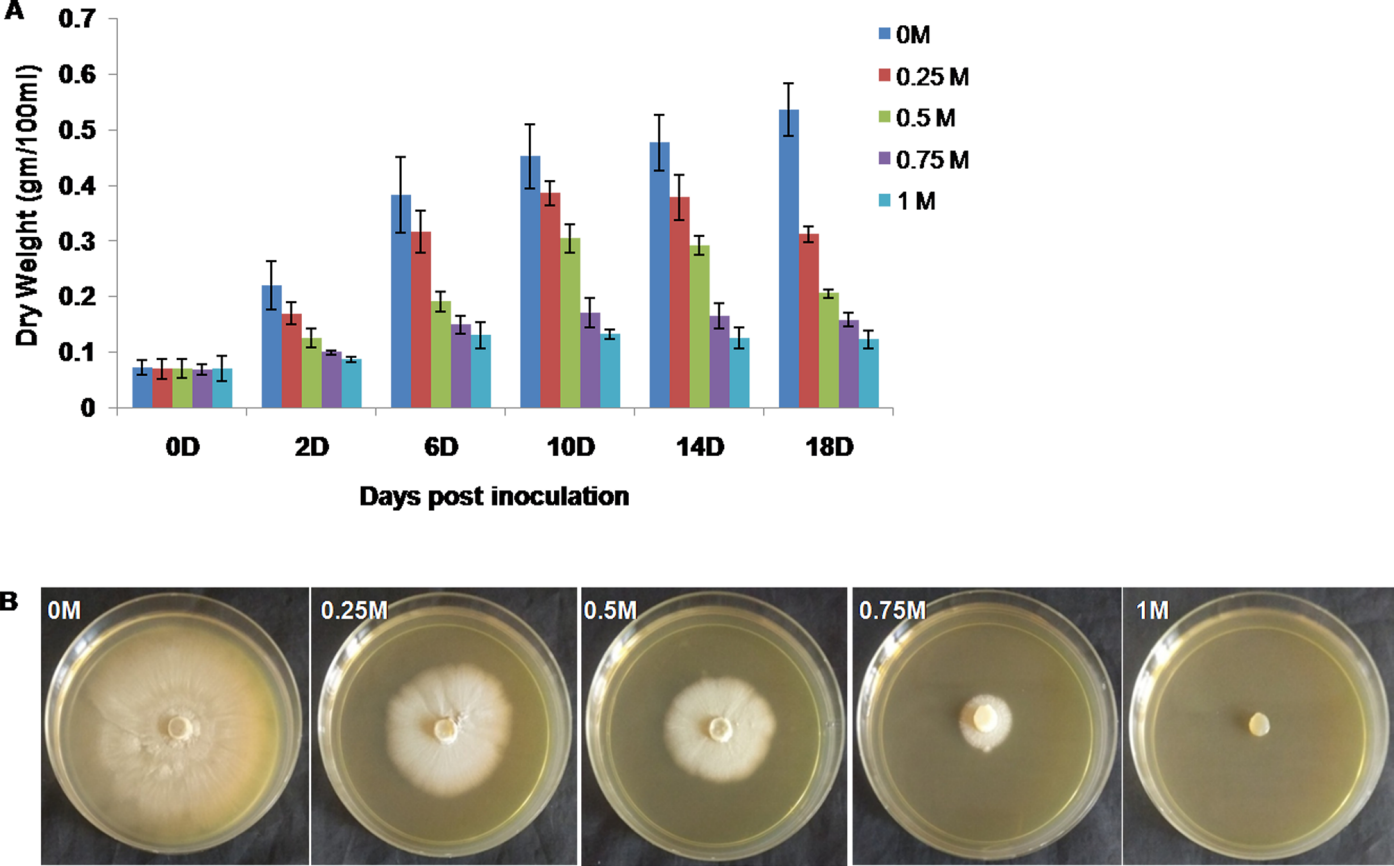
(**A**) Growth of *P. indica* under various NaCl concentrations over a different time interval. Fungal dry weights were measured at 0, 2, 6, 10, 14, and 18 days after treatment with salt (0 M, 0.25 M, 0.5 M, 0.75 M, and 1 M NaCl).Values represent means with ± standard deviations (SD) of three replicates. (**B**. Stress sensitivity of *P. indica* grown under different salt concentrations on Kaefer’s agar medium for 10 days. Each panel shows different salt treatment, i.e. 0 M, 0.25 M, 0.5 M, 0.75 M, and 1 M NaCl.

The biomass of *P. indica* was assessed under salt treatment. We observed a significant increase in dry weight (DW) of *P. indica* at 0 M, 0.25 M and 0.5 M NaCl concentrations with increasing time points i.e. 2, 6, 10, and 14 days. After 14 days there was a sharp decrease in dry weight at 0.25 M, and 0.5 M NaCl concentrations while it remained comparatively lower at 0.75 M and 1 M NaCl concentration at all the time points. Moreover, on the 14th day of salt stress, there were 20.5, 38.9, 65.5, and 73.6% declination in dry weight was observed at 0.25 M, 0.5 M, 0.75 M, and 1 M NaCl concentrations respectively, as compared to 0 M NaCl (Fig. 1A) (Supplementary Table S1). The salt stress sensitivity of *P. indica* mycelia growth was also observed on agar medium at given salt concentrations for 10 days (Fig. 1B). These observations indicate that salt treatments 0.5 M NaCl at day 14 appeared to be the optimal salt dose at which *P. indica* growth was moderate and showed less severity at given higher salt concentration as compared to other salt treatments.

### Effect of salt stress on lipid peroxidation

Malondialdehyde (MDA) is a biomarker of lipid peroxidation and is used to quantify the level of oxidative damage of the cell membranes. We observed that the MDA level was 1.4 and 2.2 fold higher in salt treatments 0.5 M and 1 M NaCl, respectively; as compared to the control sample (Supplementary Fig. S1) (Supplementary Table S2). We noticed that the impact of lipid peroxidation in fungal cells was relatively higher at 1 M NaCl as compared to 0.5 M NaCl treatment.

### Sequencing and de novo assembly of transcriptome

**Figure 2:**
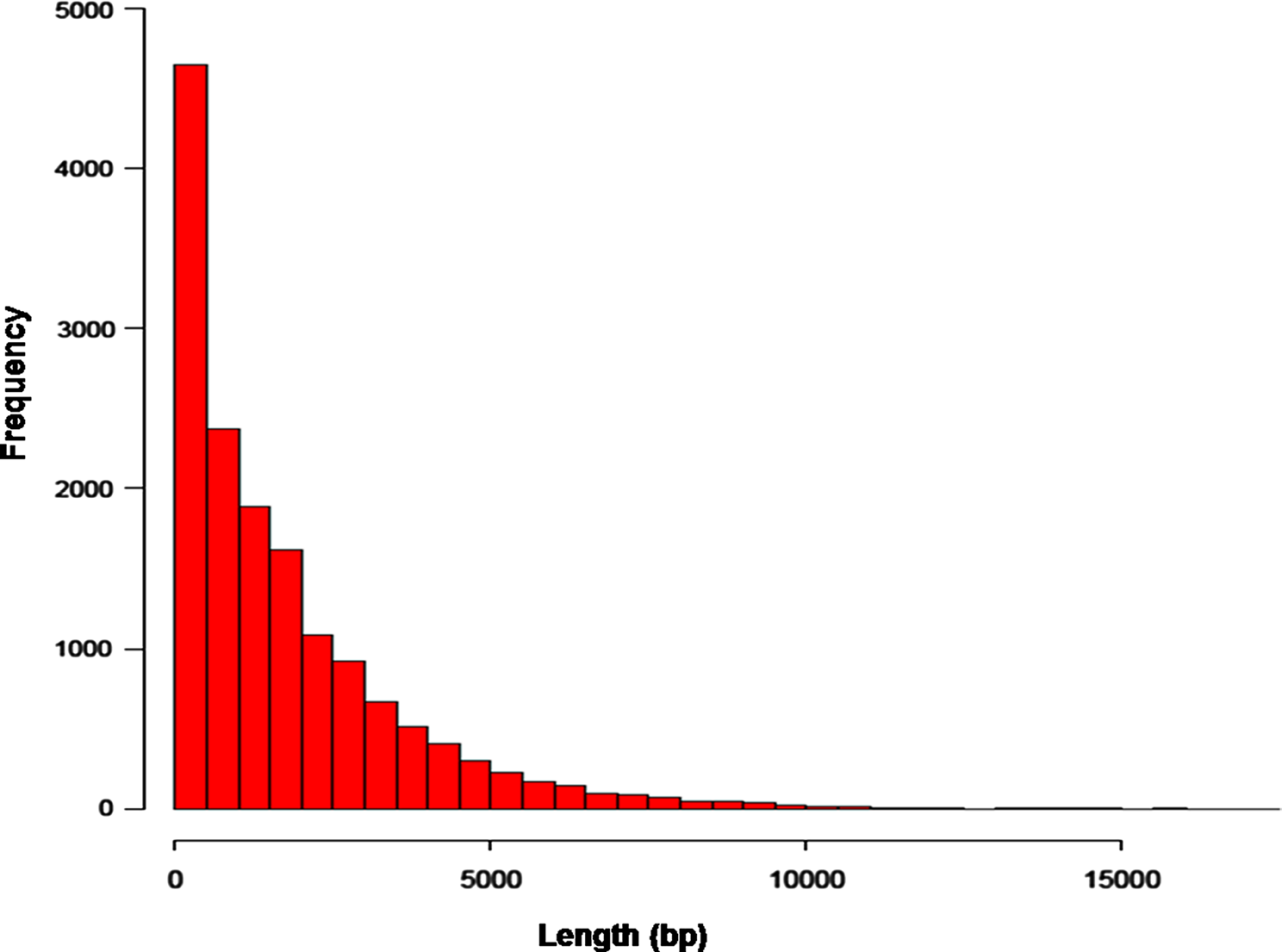
Length distribution of unigenes in the de novo assembled *P. indica* transcriptome.

Overall 78.2 million raw reads were generated from the two samples using Illumina NextSeq500. Raw reads were then subjected to trimming based on base quality score and read length, and 73.57 million clean reads were generated. Approximately 2.5% of the low-quality reads were discarded from the raw data (Table 1). Although the *P. indica* genome sequence is available in the database, it is still at the scaffold level; therefore, we decided to perform de novo assembly for the generated reads to get adequate annotation information. All the clean reads were de novo assembled into a total of 30,567 transcripts and 15,410 unigenes which were used for further analysis. The maximum transcripts length in the control and treated samples were 15,014 bp and 17,216 bp, with an average length of 1972.52 bp, and 1715.65 bp, respectively. The maximum transcripts length in the assembled unigenes was 17,216 bp with an average length of 1774.02 bp. The N50 values for the control, treated transcripts, and unigenes were 2777 bp, 3232 bp, and 3083 bp respectively. The summary of assembly statistics is shown in Table 2. Transcripts less than 500 bp in length occupied the largest proportion of all the assembled transcripts, with an average length of 1715 bp (Fig. 2).

**Table 1:** Summary statistics of *P. indica* transcriptome generated on Illumina NextSeq 500*.*

Samples	Control	Salt-treated
Total raw reads	43,514,010	34,701,482
Total clean reads	40,933,962	32,644,036
Total clean nucleotides	3,101,875,986	2,473,657,922
Max read length	76	76
Min read length	50	50
GC content% (R1, R2 processed)	53.9, 52.0	53.79, 51.8

**Table 2:** Summary of *denovo* assembly of *P.indica* transcriptome.

*denovo* Assembly Statistics			
	Control	Salt-treated	Unigene
Transcripts/Unigenes Generated	14,628	15,939	15,410
Maximum transcript/unigene length	15,014	17,216	17,216
Minimum transcript/unigene length	201	201	201
Average transcript length	1972.52	1715.65	1774.02
Total transcripts/unigene length	28,853,980	27,345,796	27,337,692
Transcripts/unigene > 200 b	14,628	15,939	–
Transcripts/unigene > 500 b	11,168	11,566	10,761
Transcripts/unigene > 1 Kb	8928	8959	8387
Transcripts/unigene > 10 Kb	67	43	62
Transcripts/unigene > 100 Kb	0	0	0
n50 value	2777	3232	3083
Number of reads used	39,910,613	31,958,512	–
Total number of reads	40,933,962	32,644,036	–
Percentage of reads used	97.5	97.9	–

### Functional annotation and characterization of *P. indica* transcripts:

**Figure 3:**
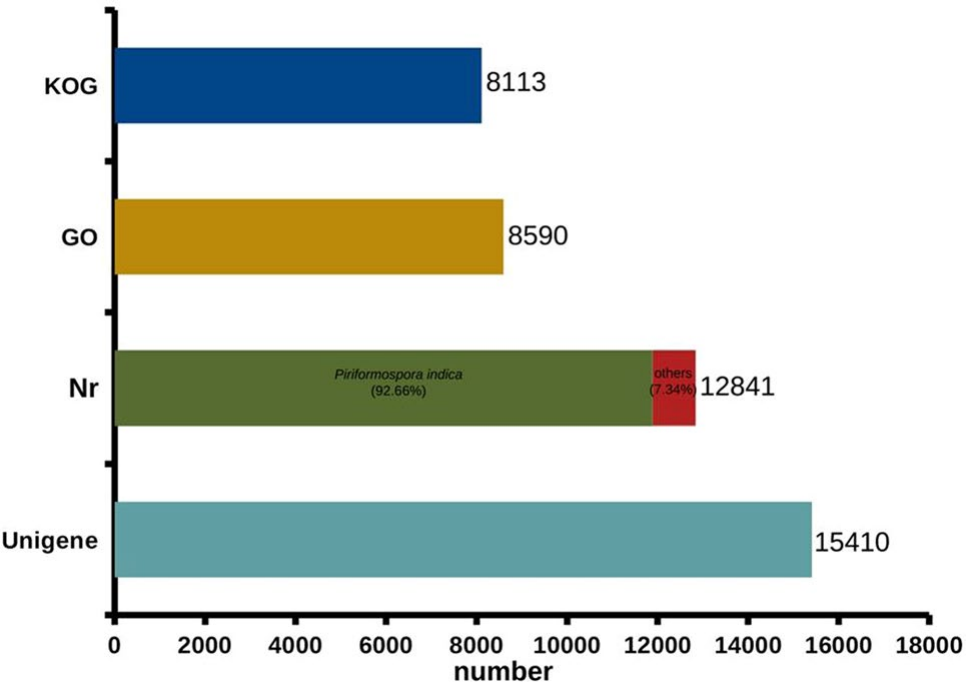
Functional characteristics of homology search of *P. indica* unigenes. Assembled unigenes were mapped against non-redundant (nr), gene ontology (GO), and EuKaryotic Orthologous Groups (KOG) database. Total 12,841 unigenes were mapped to their homologous nr protein sequences out of which 92.65% (11,898) were matched to *P. indica*. A total of 8590 unigenes were assigned to different GO terms and 8113 unigenes were mapped different KOG classes.

It is important to gather detailed functional information to understand various metabolic processes. To obtain information on the putative function of identified transcripts, the nucleotide sequences of all transcripts were compared to the NCBI non-redundant (nr) database and the Uniprot database using BLASTX. A total of 15,410 unigenes were annotated against the non-redundant (nr) protein database. Overall 12,841 (83.32%) unigenes were mapped to their homologous nr protein sequences, out of which 92.66% (11,898) were matched to *P. indica* and 7.34% (943) were matched to other species. Further, the unigenes were annotated for gene ontology and orthologous groups. Around 8590 (55.74%) unigenes were assigned to different GO terms categorized under biological process (BP; 4785), cellular components (CC; 2938), and molecular function (MF; 6234) (Fig. 3). In the BP category, metabolic process, cellular process, and biological regulation were highly represented. In the CC category, cell, cell part, and membrane were dominant. Binding and catalytic activity were most represented in the MF category (Supplementary Fig. S2). KOG classification for unigenes was performed based on the available KOG information for *P. indica* and 8113 (52.64%) unigenes were mapped to different KOG classes (Fig. 3).

### Analysis of differentially expressed genes

**Figure 4:**
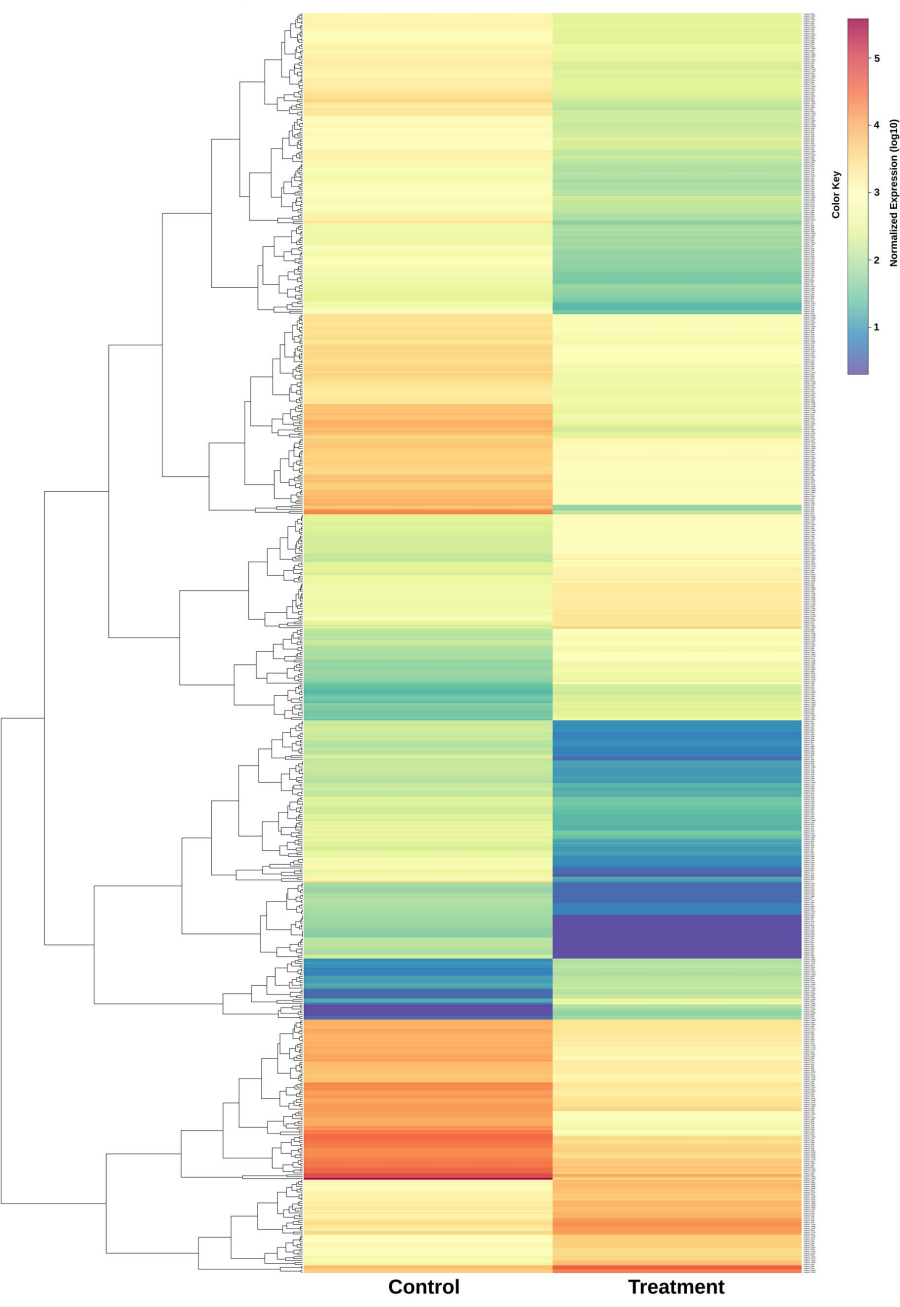
Heatmap and hierarchical cluster analysis of differentially expressed genes based on the expression pattern of 661 DEGs derived from the control (0 M NaCl) and salt-treated (0.5 M NaCl) *P. indica* 14 days after treatment. The color scale represents the expression of DEGs in terms of normalized expression of DEGs between control and salt-treated samples. Tool: gplots package in R (3.1.1).

**Figure 5:**
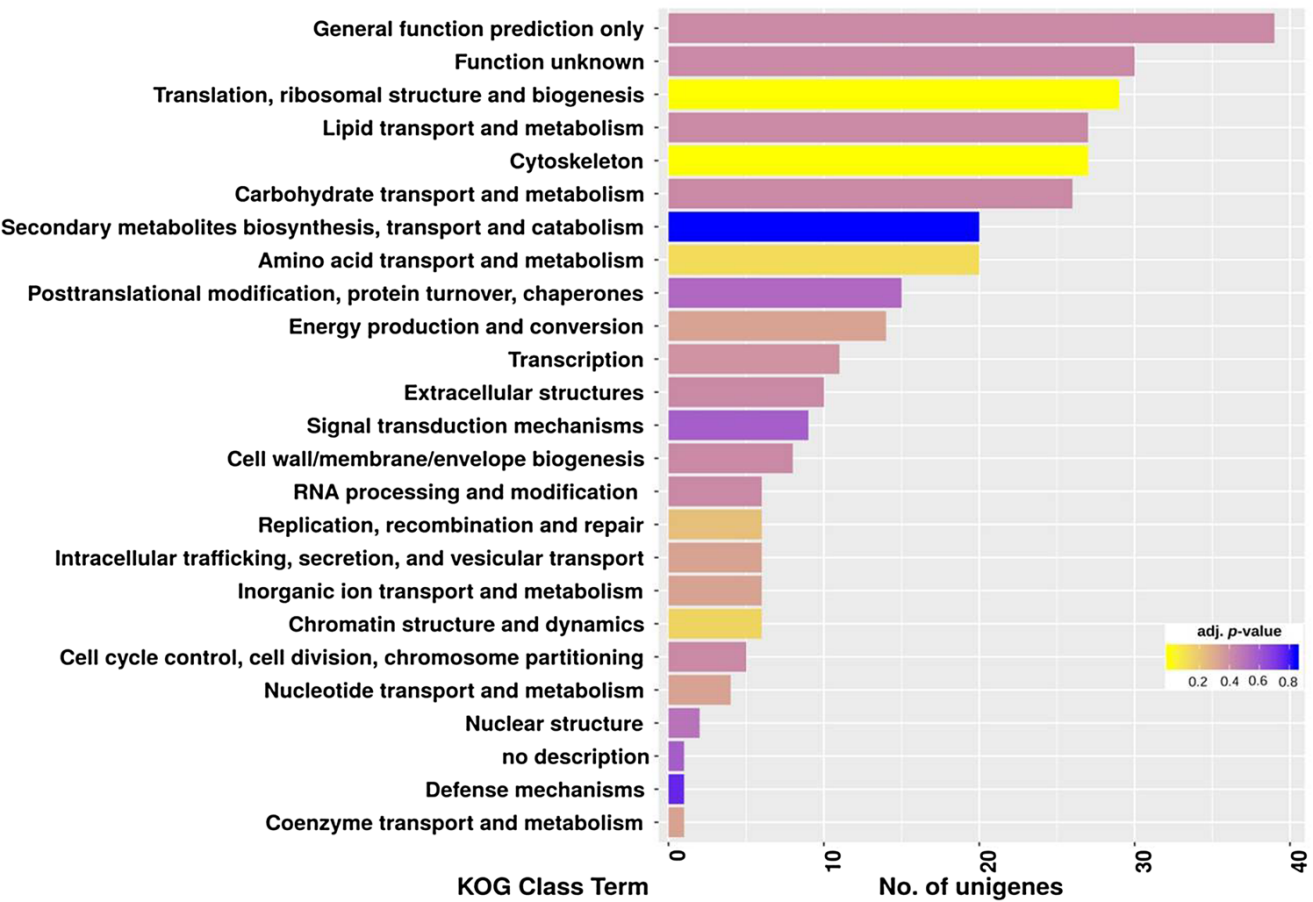
EuKaryotic Orthologous Groups (KOG) class enrichment analysis for *P. indica* DEGs under salt stress. Bars represent the number of unigenes for each KOG class (y-axis) found in the transcriptome. The significance level represented is based on adjusted *p*-value i.e. padj < 0.01.

The reads for both samples were separately aligned to de novo assembled unigene sequences and read count profiles were generated to obtain expression abundance of genes. Of the 15,410 unigenes, a total of 13,461 genes were differentially expressed between control and salt-stressed *P. indica*, among which 661 were significantly differentially expressed (*p* < 0.05) including 189 upregulated and 472 downregulated unigenes in response to salt stress. (Fig. 4) (Supplementary File S). A total 329 DEGs were assigned to KOG information that is categorized into 5 main KOG groups among which 123 unigenes belong to metabolism, 78 unigenes to cellular processes and signaling, 58 unigenes to information storage and processing, 69 unigenes to poorly characterized, and 1 unigene belong to an undefined group (Supplementary Fig. S3). KOG enrichment analysis was performed to identify significant KOG classes. DEGs were assigned to 5 major groups and 25 KOG terms, however, only two KOG classes i.e. Translation, ribosomal structure and biogenesis, and cytoskeleton were found as the most significant at adjusted *p*-value < 0.01 (Fig. 5) (Supplementary Table S3) (Supplementary File S). Next, 116 unigenes were mapped to 12 different KEGG pathway classes which are mainly related to the metabolic and regulatory pathway category (Supplementary File S).

**Figure 6:**
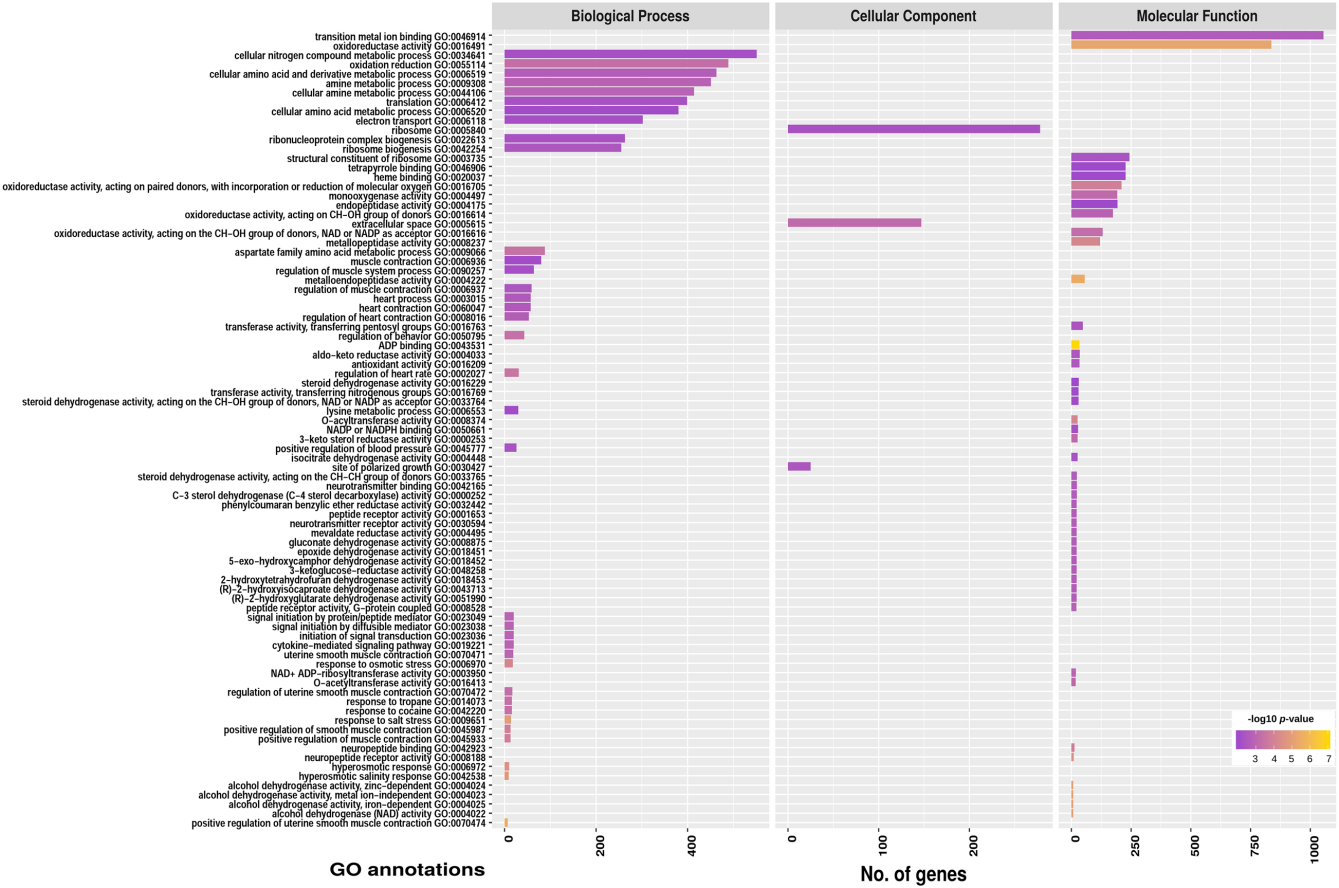
GO enrichment of DEGs of *P. indica* transcriptome under salt stress. The x-axis represents the number of unigenes involved in different GO terms. GO term plots represent the number of genes in each GO id at FDR < 0.05 and *p*-value < 0.01. Tool: ggplot2 package R v3.3.3.

To further evaluate the *P. indica* transcriptome and reinforce the efficacy of the gene annotation, the differentially expressed unigenes were assigned to singular enrichment analysis of GO terms. A total of 363 unigenes were assigned to 85 functional categories in GO enrichment analysis (Fig. 6) (Supplementary Fig. S4). The most significant GO terms in the BP category were positive regulation of uterine smooth muscle contraction, hyperosmotic salinity response, and response to salt stress. In the CC category, extracellular space, site of polarized growth, and ribosome were significantly enriched whereas ADP binding, alcohol dehydrogenase (NAD) activity, metalloendopeptidase activity, and oxidoreductase activity were most enriched in the MF category (Supplementary File S). Unfortunately, there were still many genes showing differential expression annotated as “hypothetical protein with unknown functions” (Supplementary File S). This indicates that *P. indica* might recruit some unknown players to participate in the salt stress mechanism. Therefore, it is imperative to investigate the potential function of these genes.

### Salt-responsive DEGs related to transcription factors and transporter proteins

Transcription factors (TFs) play important roles in response to environmental stimuli including salt stress. A total of 92 unigenes related to transcription factors were shown differential expression of which majority were related to GATA-4/5/6 transcription factors, helicase-like transcription factors with DEAD-box superfamily, and HOX domain related to homeobox family. However, only 5 DEGs were significant with 1 up and 4 downregulated expressions. The gene encoding putative transcription factor 5qNCA was shown upregulation while genes encoding transcription factor with HOX domain, HMG-box, putative HALR/MLL3, and helicase-like transcription factor related to DEAD-box superfamily were downregulated (Supplementary Table S4).

Next, a total of 193 hits were obtained as transporter/channel proteins upon the BLAST analysis. Out of which only 18 were significant with 9 up and 9 downregulated DEGs. Genes related to major facilitator superfamily, phosphate transporter, voltage-gated shaker-like K^+^ channel, Ca^2+^ transporting ATPase, and divalent cation transporter were among the upregulated DEGs. On the other hand, gene encoding tartrate transporter, high-affinity glucose transporter, Ca^2+^-modulated nonselective cation channel, H^+^/oligopeptide symporter, voltage-gated shaker-like K^+^ channel, and transporter belongs to major facilitator superfamily were downregulated (Supplementary Table S4).

### Identification of DEGs related to ergosterol biosynthesis

Genes associated with the steroid biosynthetic pathway were observed to be differentially expressed in our data*.* The ergosterol biosynthesis involves about 20 enzymes including the synthesis of squalene from mevalonate^31^. A total of four genes involved in ergosterol biosynthesis and mevalonate pathways were identified with higher expression in *P. indica* in response to salt stress. Gene coding for hydroxymethylglutaryl-CoA synthase (unigene_13355) that involve in the conversion of acetyl-CoA to HMG-CoA in mevalonate pathways was upregulated by 3.0 fold. Genes involved in the downstream pathway of ergosterol biosynthesis such as C-22 sterol desaturase (*ERG5*, unigene_3108), delta (24)-sterol c-methyltransferase (*ERG6*, unigene_8750), and 3-keto sterol reductase (*ERG27*, unigene_10637) were also upregulated by 2.7, 2.2, and 2.7 fold change, respectively (Supplementary Table S5). Apart from that, gene encoding lanosterol synthase that involves in sterol biosynthesis was also induced.

### DEGs involved in cell wall integrity and signal transduction

The osmotic imbalance perturbs normal cell wall integrity (CWI) and architecture. Several transcripts related to cell wall biogenesis and those involved in the CWI pathway were differentially expressed in response to salt stress in this study. Genes encoding beta-glucosidase, glucan endo-1,3-alpha-glucosidase, and chitinase were induced whereas some transcripts related to endochitinase, endo-1,3(4)-beta-glucanase, glucan 1,3-beta-glucosidase, and alpha-glucosidase were downregulated. A gene encoding Rho1 GTPase (unigene_9456), a Rho-type Ras-related small GTPase that is involved in cell wall organization and biogenesis was found to be upregulated (log2FC 2.9) whereas another gene related to Rho3 GTP binding protein (unigene_1792) was downregulated (log2FC -4.3) (Supplementary Table S5). Several genes related to kinesin light chain (KLC) were also showed differential expression of which the majority were downregulated (Supplementary Table S5).

Further, we observed that several genes related to signal transduction were found to be upregulated i.e. a hypothetical protein encoding nucleolar GTPase/ATPase (unigene_11389, log2FC 1.9), a microtubule-associated serine/threonine kinase (unigene_3471, log2FC 2.4), a hypothetical protein kinase (unigene_10289, log2FC 4.7), two hypothetical protein PIIN_02443 related to diacylglycerol kinase ATP (unigene_4146 and unigene_11878, log2FC 2.2), and a GTPase Rho1 protein (unigene_9456, log2FC 2.9) (Supplementary Table S5).

### Identification of oxidative stress and other stress-related genes

Changes in gene expression involving oxidative stress alleviation have been observed in response to high salinity in halotolerant fungi^32^. In this study, two genes involved in oxidative stress such as thioredoxin peroxidase (unigene_2182, log2FC 2.6) and nuclear protein *FAP7* (Factor Activating Pos 9, unigene_8122, log2FC 2.1) were induced (Supplementary Table S5). Genes encoding glutamate decarboxylase (unigene_13081 and unigene_3774) that involve in gamma-aminobutyrate (GABA) synthesis were also upregulated (log2FC 2 and 2.6, respectively). However, genes encoding glutathione S-transferase, glutathione synthase, thioredoxin, NADPH: quinone reductase, and several other oxidoreductases were downregulated, indicating the strict regulation of the oxidative stress pathway in response to salt stress. Genes involved in the electron transport chain and energy productions were also detected, among which ubiquinol-cytochrome c reductase was upregulated (unigene_9704, log2FC 4.8) whereas genes related to cytochrome c oxidase and cytochrome b were downregulated.

Several genes related to the cytochrome P450 family were also identified; among them, two (unigene_11721 and unigene_12293) were upregulated that are involved in secondary metabolites biosynthesis and lipid transport and metabolism. Three flavin-containing monooxygenases (unigene_5372, unigene_12730, and unigene_12731) were also found to be upregulated in response to salt stress. Stress exposure leads to the activation of many heat shock proteins (HSP/chaperon) and metalloproteases. In this study, we found that two genes encoding aspartyl protease (unigene_13173) and serine proteinase inhibitors (unigene_9129) were induced. Interestingly, all the heat shock proteins were shown downregulation in response to salt stress (Supplementary Table S5).

### DEGs involved in other processes related to salt adaptation

Many genes involved in various cellular metabolic pathways were also expressed in response to the high salt condition. Genes involved in amino acid metabolism, nucleotide transport and metabolism, nitrogen metabolism, lipid transport and metabolism, and starch and sucrose metabolism were differentially expressed in *P. indica* transcriptome. We observed that genes encoding spermidine synthase, sorbitol dehydrogenase, UDP-glucose 6-dehydrogenase, carbonate dehydratase, alpha-isopropylmalate isomerase, FOL3-dihydrofolate synthetase, SLC1-1-acyl-sn-glycerol-3-phosphate acyltransferase, choline dehydrogenase, Pirin, and 2-deoxy-D-gluconate 3-dehydrogenase were overexpressed under salt stress. Further, genes involved in “chromatin structure and dynamics”, “RNA processing and modification”, “replication, recombination and repair”, and “translation, ribosomal structure and biogenesis” such as histone-lysine N-methyltransferase, meiotic recombination protein rec8, RFC3-DNA replication factor C, RNA-directed DNA polymerase, ribonuclease H1, and several Notchless-like WD40 repeat-containing proteins were highly induced (Supplementary Table S5) (Supplementary File S).

### Validation of gene expression using Real-time quantitative PCR

**Figure 7:**
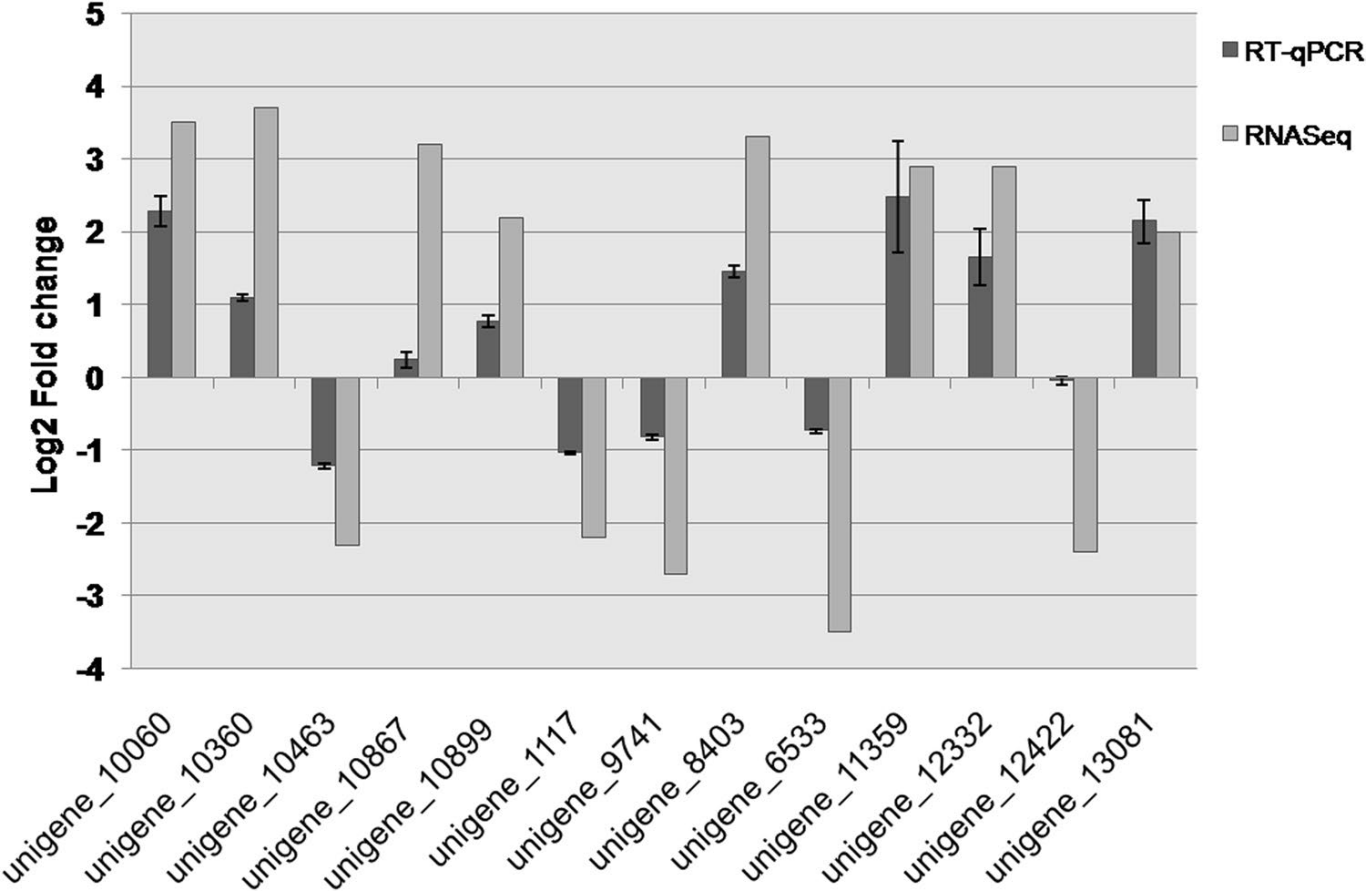
Validation of RNA-Seq data using RT-qPCR. Thirteen representative genes were selected to validate the RNA-Seq data by RT-qPCR. The dark grey bars represent mean values of log2-fold change obtained from three replicates of RT-qPCR with error bars representing ± SD and the light grey bars represent the RNA-Seq data.

A total of 13 salt-responsive genes were selected to perform real-time quantitative PCR (RT-qPCR) to confirm and validate the expression levels of unigenes which were identified in transcriptome data. We used *PiTEF* as a reference gene. The selected genes showed similar expression patterns as observed in our transcriptome analysis. The results of RT-qPCR were consistent with the RNA-Seq analysis, confirming the reliability of transcriptome analysis (Fig. 7) (Supplementary Table S6).

## Discussion

The endosymbiont *P. indica* has gained considerable attention since the past decade for its ability to ameliorate biotic and abiotic stress tolerance in the host plants. However, the transcriptional responses of *P. indica* to high salt stress have yet to be delineated. The recent advances in high-throughput sequencing technologies, for example, RNA-Seq and the genome sequencing of *P. indica* have helped tremendously to provide a global view of the *P. indica* transcriptome^31,33^.

Salinity stress can greatly affect the growth of a living organism mainly due to the accumulation of ROS and by-products of lipid peroxidation^34^. In our preliminary results, *P. indica* showed reduced biomass and higher MDA level with increasing salt doses. However, the extent of weight loss and lipid peroxidation were less severe at 0.5 M NaCl as opposite to 1 M NaCl which indicates that *P. indica* employs certain strategies to cope with the negative impact of high salinity.

In this study, the RNA-Seq experiment of *P. indica* provided a comprehensive detail of gene expression differences where the number of downregulated genes was higher in response to salt stress than the upregulated genes. The 73.57 million clean reads of generated sequence data possibly provided adequate coverage of the 25 Mb genome of the root symbiont *P. indica*. To achieve a high percentage of annotation for the assembled transcripts, five different databases [nr/nt (GenBank), Uniprot, KEGG, KOG, and GO] were used. The de novo assembly of *P. indica* transcriptome produced a total of 15,410 unigenes, out of which 661 genes were observed to be differentially expressed in response to the high salt condition.

The GO enrichment analysis showed that the majority of DEGs were categorized under metabolic processes and molecular functions, mainly representing “response to salt stress”, “hyperosmotic salinity response”, “amino acid metabolic process”, “ADP binding”, “oxidoreductase activity”, “metallopeptidase activity”, and “monooxygenase activity” classes. These frequent functional terms reflect the importance of the regulation of cellular metabolism and gene functions involved in the stress adaptation and hyphal growth of *P. indica* in response to salinity stress. Next, the KOG enrichment revealed that the “translation”, “ribosomal structure and biogenesis”, “cytoskeleton”, and “metabolism” were the most significant terms in *P. indica* under salinity stress suggesting the active cellular dynamics during stress.

Hypersalinity results in osmotic stress as well as ionic stress for cells which leads to higher intracellular Na^+^ accumulation that can damage membrane systems and cytosolic proteins. An efficient transport system is needed to eliminate the toxic Na^+^ ions and manage the ion homeostasis^35,36^. Transporter proteins belong to major facilitator superfamily (MFS), mirA, inorganic phosphate transporter (Pho84), voltage-gated shaker-like K^+^ channel, and divalent cation transporter were overexpressed whereas a voltage-gated shaker-like K^+^ channel, H^+^/oligopeptide symporter, Ca^2+^-modulated nonselective cation channel, and few ABC transporters were downregulated upon high salinity exposure suggesting their regulatory control over cellular ion homeostasis. The MFS belongs to a diverse group of secondary transporters that are involved in solute and metal transport in response to chemiosmotic ion gradients^37^. In *Aspergillus nidulans*, the *mirA* gene, encoding a siderophore transporter, has been characterized that helps in the uptake of iron^38^. A high-affinity membrane phosphate importer (Pho84) has been identified and characterized in *Saccharomyces cerevisiae* and *P. indica* (PiPT) which is required for cell growth and division and plays an important role in TORC1 (Target of Rapamycin Complex 1 Kinase) activation involving oxidative stress and micronutrient homeostasis^39,40^. It seems that these transporters are required to retain the cellular homeostatic balance and avoid ion toxicity in *P. indica* in addition to support the hyphal growth during salinity stress.

Transcription factors play diverse roles in the fungal developmental processes and environmental stresses^41^. Transcription factor 5qNCA containing a jmjC domain is upregulated in this study which is reported to be involved in the regulation of chromatin remodeling^42^. However, other TFs (e.g. HMG-box, Helicase-like transcription factor HLTF) that control the expression of many genes involved in developmental and metabolic pathways were downregulated in response to salt stress in our study that explains the down expression of many DEGs.

The accumulation of soluble sugars and polyamine synthesis is important in the restoration of cellular homeostasis during osmotic and salt stress. Genes related to sorbitol dehydrogenase that is involved in d-galactose and d-sorbitol catabolism and spermidine synthase were induced suggesting its importance in maintaining the homeostatic equilibrium of cells^43,44^.

Further, our transcriptome study showed that the components of the sterol biosynthesis, cell wall/membrane, chromatin structure and dynamics, and ribosome were induced under saline stress condition in *P. indica*. We observed that the genes involved in ergosterol biosynthesis were upregulated in response to high salt exposure. Sterols act as non-enzymatic protective molecules against reactive oxygen; have a strong response to salt stress and a potential target of antifungal drugs^45^. In yeast, sterols majorly contribute to the fluidity of lipid membranes and also play a vital role in vesicle formation, protein sorting, cytoskeleton organization, endocytosis, and mating^46,47^. Increased activity of 3-hydroxy-3-methylglutaryl coenzyme A reductase (HMGCR), a key regulatory enzyme in sterol biosynthesis, was observed during higher salinity stress in halophilic black yeast^48^. Previous reports showed that the erg mutants, generated by a mutation in genes of ergosterol biosynthesis (*erg 2*, *erg 3*, *erg 4*, *erg 5*, and *erg 6*) were more sensitive to salt stress than the wild type^49^. Stearoyl-CoA desaturase, an enzyme involved in the biosynthesis of fatty acids, was induced in *P. indica* under salt stress which is consistent with the earlier reports. The enzyme was speculated to be involved in maintaining the cell membrane fluidity and thereby preventing the cell membrane damage due to the excess of Na^+^ ions^50,51^.

Many genes involved in cell wall integrity and modification were differentially expressed under salt stress in this data. Activation of genes related to chitinase, beta-glucosidase, glucan endo-1,3-alpha-glucosidase, glycosyltransferase, glycoside hydrolase family 16 protein, and Rho-type GTPase indicating fungal cell wall undergoes extensive remodeling to prevent cell damage from salt stress^52^. In *S. cerevisiae,* Rho-type GTPases play an important role in the CWI signaling pathway by regulating the synthesis of cell wall glucans and chitin and involved in the crosstalk with other stress-responsive signaling pathways such as the high-osmolarity glycerol (HOG) pathway and TOR signaling^53,54^.

The increase in reactive oxygen species (ROS) level and thereby oxidative stress response can be triggered by high salinity^55^. A significant change in gene expression was observed in transcripts related to thioredoxin peroxidase, FAP7 (a predicted nucleotide kinase or nuclear protein involved in oxidative stress response), and genes related to monooxygenase in response to salt treatment. Mitochondria are directly involved in metabolic responses to osmotic stress that influences cellular energy demands. In *S. cerevisiae*, a mutation in electron transport chain activity renders cells unresponsive to osmotic shock^56,57^. In our study, genes related to ubiquinol-cytochrome c reductase, alpha-isopropylmalate isomerase (aconitase), and flavin-containing monooxygenase were shown higher fold change under high salinity which is consistent with previous studies^8,58^. However, other reducing molecules involved in respiratory chain and energy production such as NADPH: quinone reductase, cytochrome c oxidase, and cytochrome b were substantially downregulated which suggests that salt stress has an inhibitory effect on these reducing complexes^59^. The mitochondrial respiratory chain is a main source of ATP as well as ROS generation. The increased level of energy demand and resulting cellular oxidative stress in the hypersaline environment suggests a salinity-regulated mechanism relating to mitochondrial activity^32,60^. The above information indicates that fungal cells tend to reduce the mitochondrial respiratory function by suppressing the activity of its many enzymes to overcome the oxidative stress while few enzymes remain activated to meet the energy requirement of growing cells during salt stress. Additionally, the upregulation of gene encoding GABA-producing enzyme glutamate decarboxylase confirms its involvement in the tolerance against oxidative stress, germination, and hyphal development^61,62^.

We also observed the activation of genes encoding cytochrome P450 in response to salt-treatment. These proteins are required for the biosynthesis of several vital compounds such as hormones, defense-related products, and fatty acids^51^. The upregulation of cytochrome P450 during salt stress in *P. indica* might help in energy restoration, membrane repair, and fatty acid synthesis.

In filamentous fungi, microtubule-mediated transport is required for membrane and cell wall expansion and supports efficient hyphal growth^63^. Kinesin is a microtubule‐dependent enzyme and composed of two kinesin heavy chains (KHC) and two kinesin light chains (KLC). The present data demonstrated the differential expression of several genes related to KLC with mostly downregulated genes. Experimental evidence related to KLC is scarce; however few reports speculated that KLC may be an essential factor for kinesin-cargo binding^64^ or might negatively regulate the kinesin activity^65^. It is possible that the repression of KLC is required for cytoskeleton dynamics that regulate hyphal growth during stress. However, the precise function of KLC in fungi has remained undetermined. Furthermore, several genes related to WD40-repeat proteins were differentially expressed in this study. WD40-repeat proteins belong to a large protein family and participate in many biological processes including DNA replication, transcription, RNA processing, histone modification, and protein degradation^66^. A WD40-repeat protein was reported to be involved in the regulation of fungal cell differentiation and notch signaling activity during cell development^67,68^.

Moreover, genes encoding histone-lysine N-methyltransferase were also induced in response to salt stress. Histone-lysine N-methyltransferases play a crucial role in the epigenetic gene regulation and transcriptional silencing by histone methylation and chromatin modification^69^. The upregulation of these genes might be responsible for the repression of other gene expressions in this study which is possibly required to restrict the consumption of cellular energy during salt stress. Further, the activation of genes involved in central metabolic systems including glycolysis/gluconeogenesis, starch/sucrose metabolism, TCA (tricarboxylic acid cycle) cycle, glyoxylate metabolism, nucleotide metabolism, and RNA metabolism might suggest that the fungal cellular machinery actively participates in the mitigation of damage induced by hypersaline stress. It is reported that *P. indica* influences the plant metabolism and immune system through small secreted effector-like proteins during symbiosis^30^. A hypothetical protein PIIN_09643 (unigene_557) belongs to the effector family DELD was downregulated in our data in contrast to previous work^30^. On the other hand, a unigene_10360 related to glycoside hydrolase family 16 that function as plant cell wall degrading enzymes required for fungal penetration, was upregulated^30^. This suggests that *P. indica* activates different sets of gene repertoire under symbiotic and non-symbiotic phase during stress conditions.

We speculate that *P. indica* deploys mevalonate pathway via HMG-CoA synthase and CWI pathway via Rho-type GTPase to restore the membrane fluidity and integrity during salt stress. Rho-type GTPases could trigger the HOG and/or TOR pathway that are implicated in osmoadaptation, salinity stress, and cell growth, thereby attenuating the detrimental effects of salinity stress. Surprisingly, we could not detect genes related to the HOG pathway among DEGs except a unigene_12963 related to mitogen-activated protein kinase showing downregulation. The CWI pathway might also regulate the transport system and mitochondrial enzymes to sustain ionic balance and energy demands as well as to prevent further ROS generation in stressed cells.

There are a considerable number of salt-responsive DEGs in our study that are categorized as hypothetical or uncharacterized. These highly induced/repressed uncharacterized genes could have a crucial role in stress tolerance in the endophytic fungi and therefore need the proper functional assignment. Further investigation of these genes might explain the exact mechanism of the stress regulation in *P. indica*. The manipulation of *P. indica* genes such as *PiCyPA, PiPT*, and *PiHOG1* in the previous studies demonstrated the importance of the molecular study of stress tolerance and adaptive response in *P. indica* for the crop improvement during salt stress.

## Conclusions

Altogether, we conclude that *P. indica* triggers numerous salt-responsive genes with pivotal roles in stress adaptation and cellular growth during salt stress. Our results showed that genes involved in ion transport, cell wall metabolism, lipid metabolism, energy production, cytoskeleton dynamics, and signaling are particularly activated in response to salt stress. Genes identified in this study could be promising candidates for the exploitation in crop improvements. The uncharacterized DEGs in present study might resolve some unanswered questions related to fungal stress response and thus deserve further exploration. The data demonstrated here may serve as an indispensable asset for elucidating the molecular function and physiology of basidiomycetes as well as application of *P. indica* in sustainable crop production. This is the first transcriptome-wide analysis of *P. indica* against salt stress which not only provides a comprehensive transcriptome profile of cells exposed to high salt but also reinvigorates the biological insights into differentially expressed genes.

## Materials and methods

### Strain, growth conditions, and stress treatments

*P. indica* cultures (DSM11827, Deutsche Sammlungfür Mikroorganismenund Zellkulturen, DSMZ, Braunschweig, Germany) were propagated at 28 ± 2 °C in liquid/solid Kaefer’s medium^70^. The 4–5 agar discs (4 mm in diameter) of fully grown fungal mycelia was inoculated into 500 ml Erlenmeyer flasks containing 250 ml of liquid Kaefer’s medium with different concentrations of salt (NaCl) i.e. 0 M, 0.25 M, 0.5 M, 0.75 M, and 1 M NaCl. Fungal culture was incubated at 28 ± 2 ºC with constant shaking at 150 rev/min in the metabolic shaker and growth patterns were recorded. *P. indica* was also grown on the 90 mm Petri plates containing solid Kaefer’s medium supplemented with different salt concentrations as above for 10 days. The growth of *P. indica* was measured in terms of dry weight by collecting fungal hyphae at 0, 2, 6, 10, 14, and 18 days post treatment. The fungal hyphae treated with 0 M and 0.5 M NaCl at 14 days were sampled and stored at − 80 ºC before RNA isolation.

### Determination of MDA content

*P. indica* mycelium was treated with 0 M, 0.5 M, and 1 M NaCl for 1 week in Kaefer’s medium as described above. The MDA content was measured as described previously by Heath and Packer method^71^. Fungal mycelia (0.1 g) was homogenized in 0.5 ml of 0.1% (w/v) TCA (tricarboxylic acid) and centrifuged at 10,000 × *g* at 4º C for 10 min. The 0.5 ml of supernatant was added to 1.5 ml of 20% (w/v) TCA and 0.5% (w/v) TBA (thiobarbituric acid). The mixture was incubated at 95º C for 25 min and then placed on ice to stop the reaction. The absorbance of the solution was measured at 532 nm and 600 nm in PerkinElmer Lambda 40 Spectrophotometer. The MDA content was calculated by subtracting OD_600_ values from OD_532_ values and using Lambert–Beer law with an extinction coefficient of 155 mM^−1^ cm^−1^. The results were represented as nmol fresh weight g^-1^. The experiment was performed in three or more replicates.

### RNA extraction, library construction, and sequencing

The total RNA of selected treatments from different replicates was extracted and pooled together using Qiagen following the manufacturer’s instructions. The RNA quality was assessed using 1% agarose gel, NanoDrop 1000 spectrophotometer (Thermo Scientific, USA), and a 2100 Bioanalyzer RNA Nano chip (Agilent Technologies, GmbH, Berlin, Germany). Total RNA was treated with RNase-free DNaseI according to the manufacturer’s recommendation (Ambion, USA). The ribosomal RNA was removed using Ribo-ZeroTM rRNA Removal Kit according to the manufacturer’s protocol (Epicentre, USA). RNA with RIN value greater than 9.0 was used for transcriptome library construction. The cDNA libraries were constructed with TruSeq RNA Sample Prep Kit (Illumina, USA) according to the manufacturer’s protocol (Illumina Inc., CA, USA). After quality assessment with Agilent 2100, each cDNA library was sequenced with Illumina NextSeq500 platform according to the manufacturer’s instructions (Illumina, USA).

### Preprocessing and de novo assembly

The 76 bp paired-end raw reads, generated from Illumina NextSeq500, were preprocessed and adapter sequences were removed using In-house Perl scripts which: (a) remove low-quality bases with Phred score < 20, (b) remove ambiguous base ‘N’, and (c) discard short reads with length < 25 bp. Further, the high quality filtered reads were de novo assembled using Trinity (trinityrnaseq20140413p1) and bowtie (bowtie-1.1.0) with default parameters to construct transcripts. Final clustering of transcripts was conducted using the Cluster Database at High Identity with Tolerance (CD-HIT) EST suits (cd-hit-v4.6.1) with a minimum similarity cut-off of 90% to generate the non-redundant transcripts used for further analysis.

### Differential expression analysis and functional annotation of transcripts/genes

Gene expression level was calculated by mean normalized counts by using the DeSeq tool^72^ which provides methods to test for differential expression by using negative binomial distribution and shrinkage estimator for the distribution’s variance. The transcript abundance estimation and their differential expression were analyzed between salt-treated and control sample libraries. Transcripts/unigenes identified with the false discovery rate (FDR) corrected *p*-value < 0.05 by using the Benjamini–Hochberg procedure were considered as significantly expressed between two samples. The differentially expressed unigenes were considered to be upregulated and downregulated if normalized fold change (log2 fold change or log2FC) was > onefold with *p*-value < 0.05.

Moreover, for functional annotation, the non-redundant transcripts were searched against the NCBI’s non-redundant (nr) and Uniprot database using the BLASTX algorithm with e-value ≤ 10^–5^. We also mapped unigenes to the GO database using Blast2GO (http://www.blast2go.de) for GO functional classification. Singular enrichment analysis (SEA) of GO terms for differentially expressed unigenes was performed using the custom tool of AgriGO v2.0 at FDR < 0.05 and *p*-value < 0.01. EuKaryotic Orthologous Groups (KOG) was used to assign different KOG classes for unigenes by using R package version 1.1 KOGMWU **(**https://CRAN.R-project.org/package=KOGMWU**)**. Mann–Whitney U test was performed to identify significant KOG class using the ranking-based method.

### Real-time PCR validation

The expression of identified unigenes by RNA-seq was validated using RT-qPCR. For this, we selected 13 unigenes showing differential expression between control and salt-treated samples. The same samples which were utilized for RNA-seq were used for the RT-qPCR analysis. The total RNA was reverse transcribed into cDNA using M-MLV Reverse Transcription Kit (Promega, USA) according to the manufacturer’s instructions. Gene-specific primers (Supplementary Table S7) for RT-qPCR were designed with help of integrated DNA technologies (IDT) software (https://sg.idtdna.com/primerquest/home/index). A 10 µl reaction was carried out on the Roche real-time PCR system (Roche, Switzerland) with the absolute SYBR Green qPCR Kit Master Mix according to the manufacturer’s instructions. The cycle threshold value (CT) was determined and differential expression was calculated using the 2^−ΔΔCT^ method^73^ with the TEF gene (*PiTEF*) of *P. indica* used as an endogenous reference. Each experiment was performed in triplicate.

### Statistical analyses

The statistical tests were computed with Microsoft excel and SPSS (version 21) and the significance of differences between data sets was evaluated using ANOVA and t-test.

## Supplementary Information

Supplementary Information.

Supplementary Information.

Supplementary Information.

Supplementary Information.

Supplementary Information.

Supplementary Information.

Supplementary Information.

Supplementary Tables.

Supplementary Tables.

Supplementary Legends.

## Data Availability

The results of the RNA sequencing experiments have been submitted to the NCBI SRA database under the accession number PRJNA540540.
