# Supplementary figures and images for: A high-throughput RNA-Seq approach to elucidate the transcriptional response of Piriformospora indica to high salt stress
Source: Sci Rep. 2021 Feb 18;11:4129. doi: 10.1038/s41598-021-82136-0 (PMC7893156; doi:10.1038/s41598-021-82136-0)

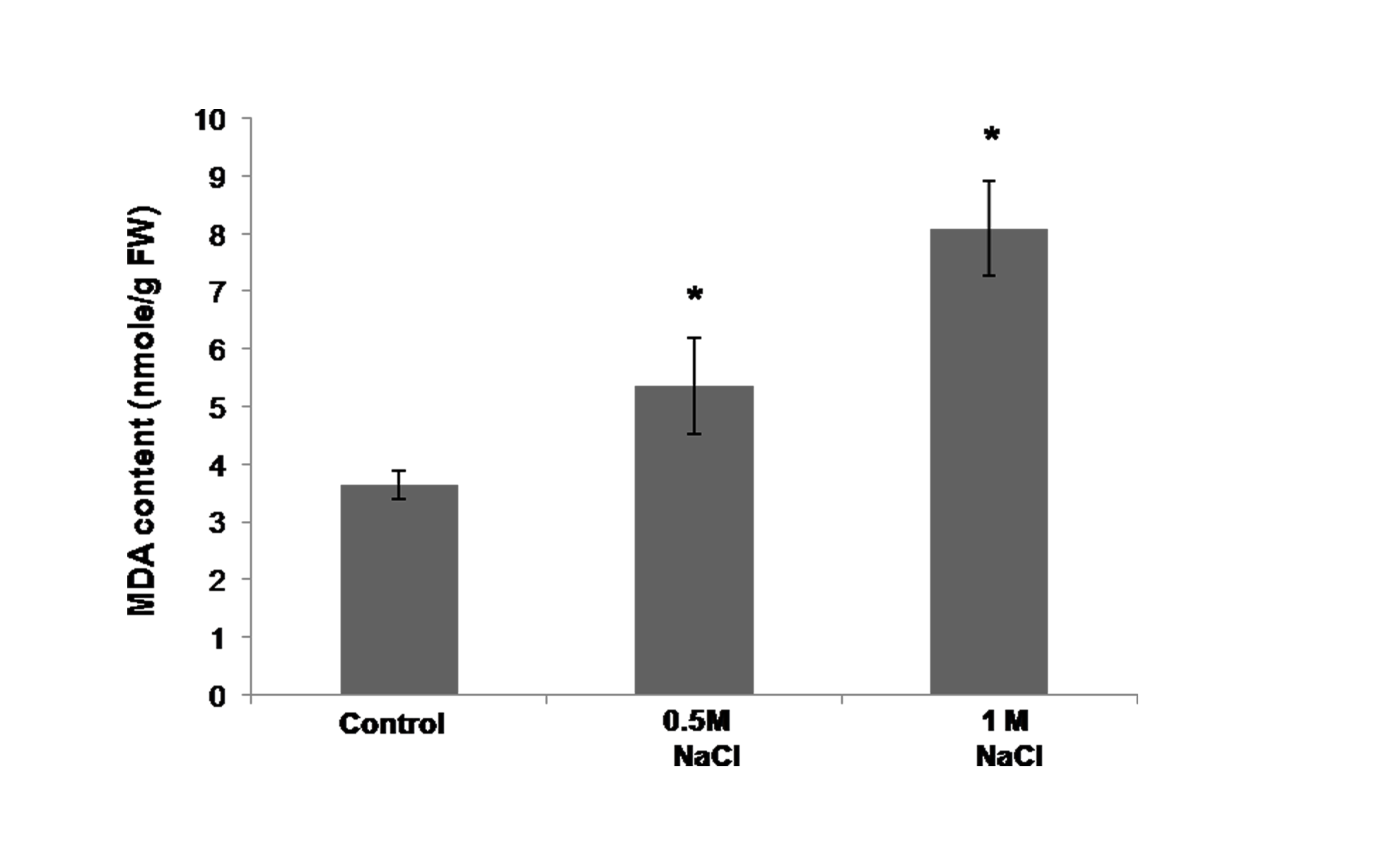

Supplement: Supplementary file 2 — Supplementary Information. [file 41598_2021_82136_MOESM2_ESM.tif]

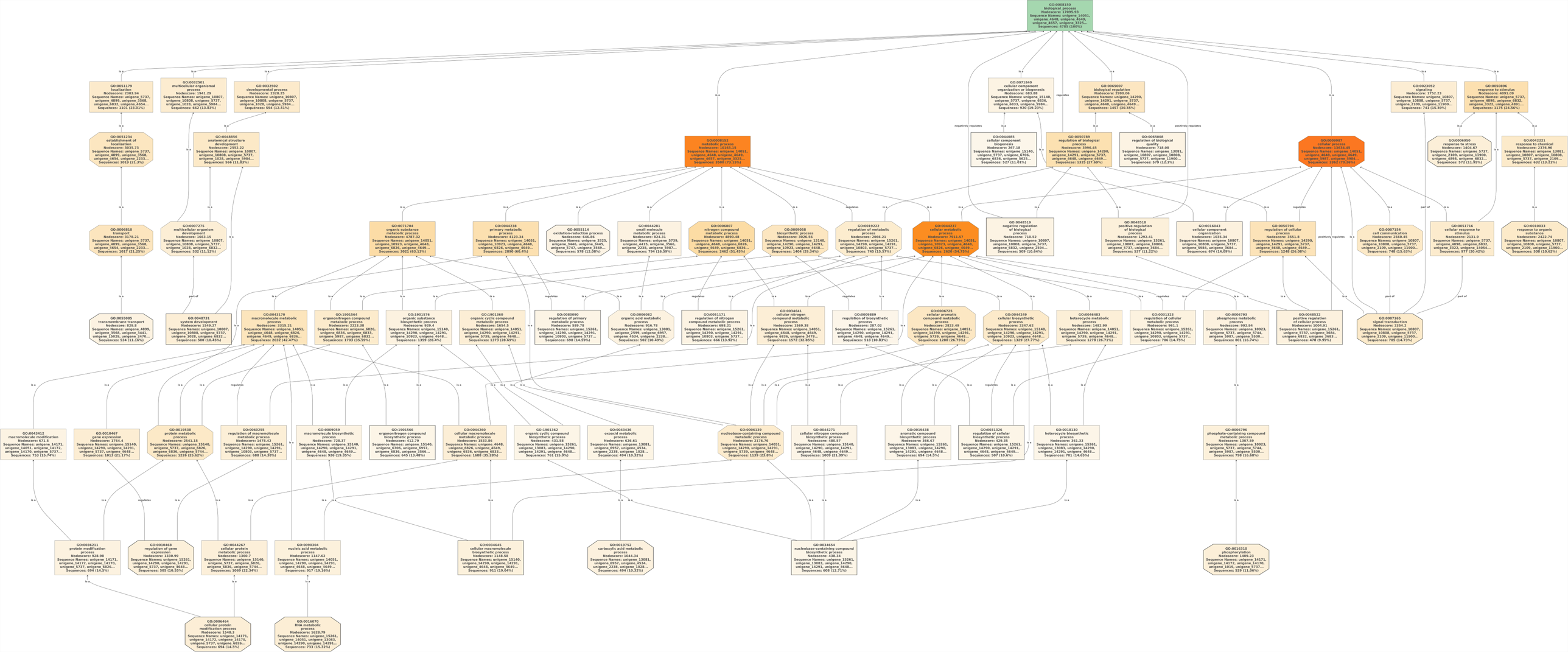

Supplement: Supplementary file 3 — Supplementary Information. [file 41598_2021_82136_MOESM3_ESM.tif]

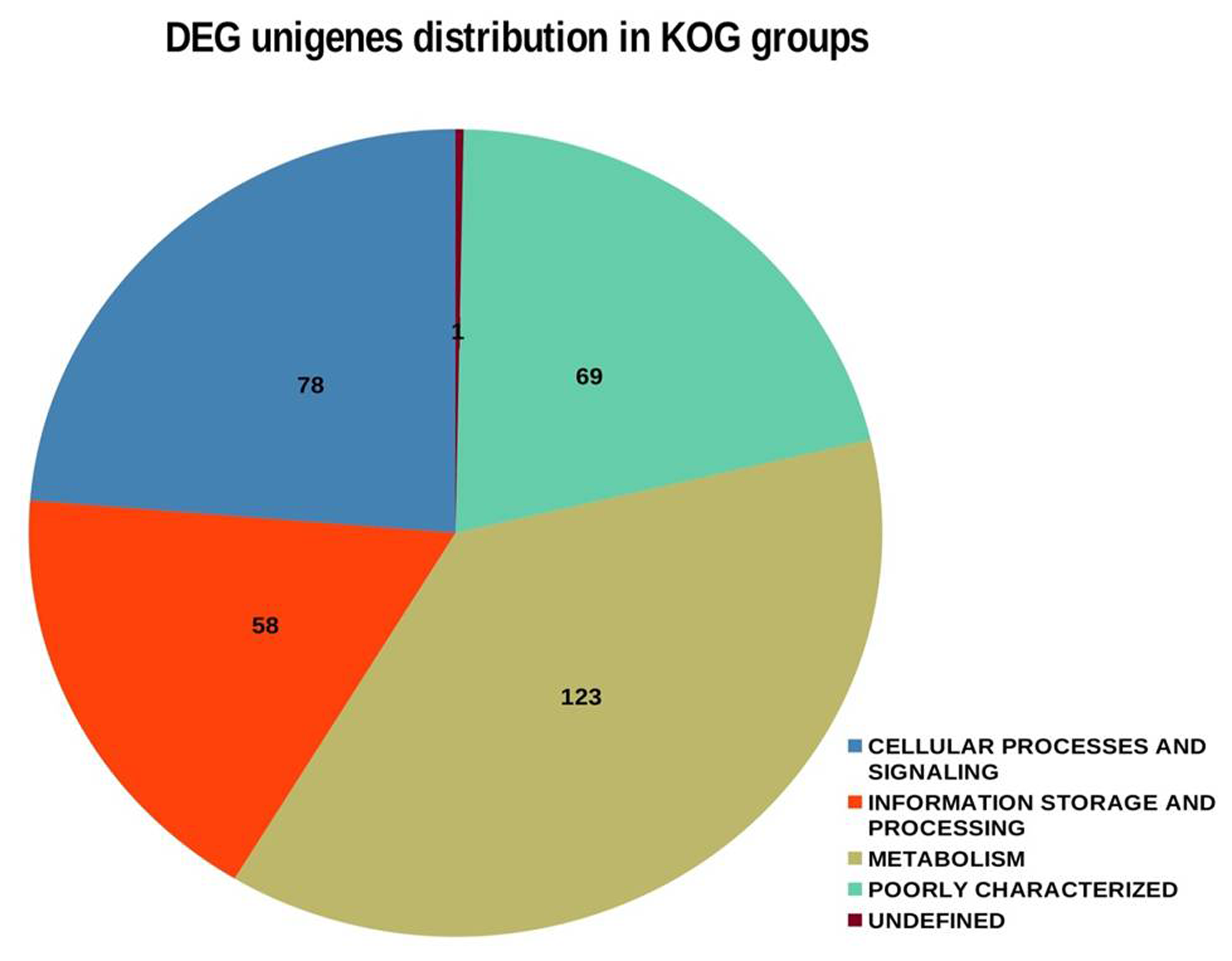

Supplement: Supplementary file 6 — Supplementary Information. [file 41598_2021_82136_MOESM6_ESM.tif]

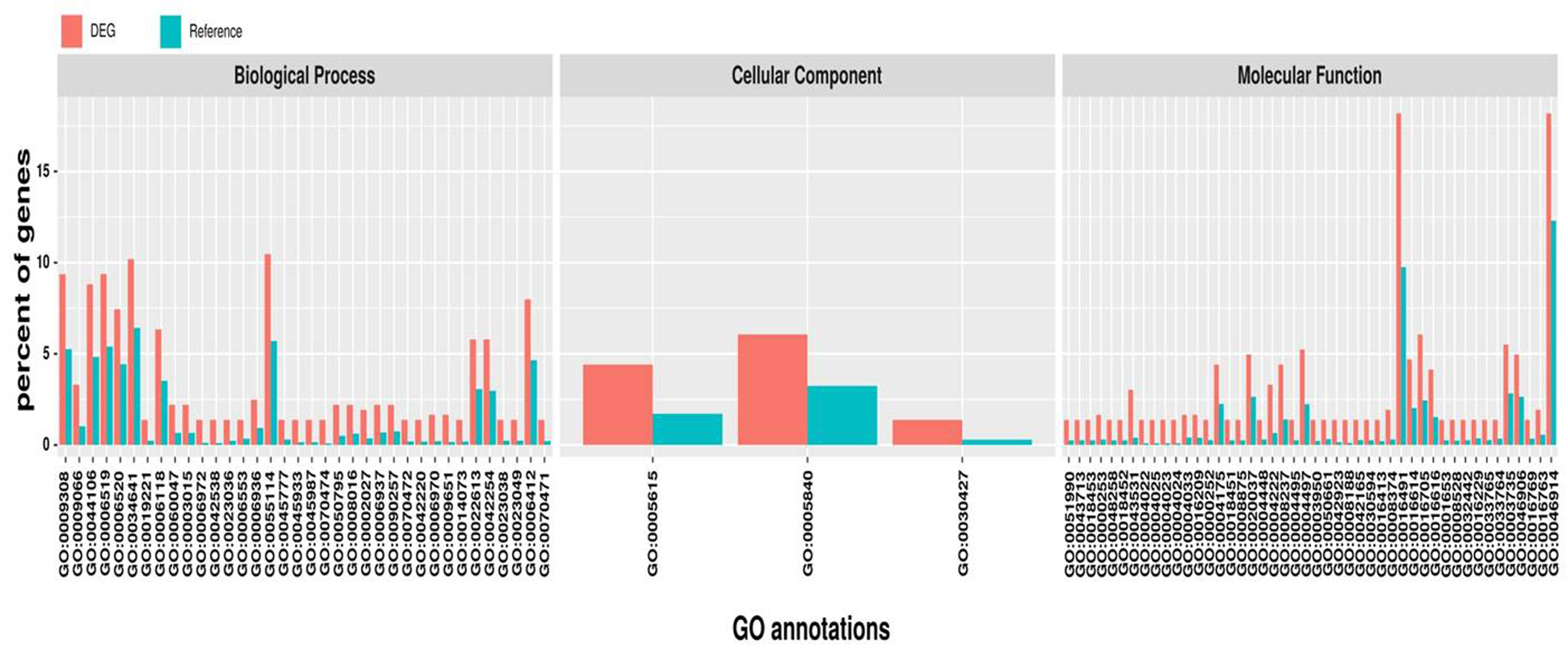

Supplement: Supplementary file 7 — Supplementary Information. [file 41598_2021_82136_MOESM7_ESM.tif]
