# Supplementary material for: A high-throughput RNA-Seq approach to elucidate the transcriptional response of Piriformospora indica to high salt stress
Source: Sci Rep. 2021 Feb 18;11:4129. doi: 10.1038/s41598-021-82136-0 (PMC7893156; doi:10.1038/s41598-021-82136-0)
Supplement: Supplementary file 8 — Supplementary Tables. [file 41598_2021_82136_MOESM8_ESM.docx]

Supplementary information:

**Title: A high-throughput RNA-Seq approach to elucidate the transcriptional response of *Piriformospora indica* to high salt stress.**

**Authors: Nivedita^1^, Abdul Rawoof^2^, Nirala Ramchiary^2^, *Malik Z. Abdin^1^**

**Affiliation:** 1. Department of Biotechnology, Jamia Hamdard, New Delhi, India.

2. School of Life sciences, Jawaharlal Nehru University, New Delhi, India.

*Corresponding author: Malik Z. Abdin

Department of Biotechnology, Jamia Hamdard, New Delhi, India

Email: mzabdin@jamiahamdard.ac.in, Ph: +919818462060

Table S1. Dry weight of *P. indica* treated with different salt (NaCl) concentrations at different time periods.

| Dry weight (gm) | | | | | | |
| --- | --- | --- | --- | --- | --- | --- |
| NaCl | **0D** | **2D** | **6D** | **10D** | **14D** | **18D** |
| 0M | 0.072333  ±0.012662 | 0.219333  ± 0.043753 | 0.382333  ± 0.06853 | 0.451667  ±0.057709 | 0.476333  ±0.049541 | 0.535667  ±0.046929 |
| 0.25 M | 0.069667  ±0.018583 | 0.169  ±0.019975 | 0.316  ±0.038354 | 0.386  ±0.021378 | 0.378333  ±0.040278 | 0.312  ±0.014 |
| 0.5 M | 0.070667  ±0.017156 | 0.125  ±0.016703 | 0.190667  ±0.017673 | 0.303667  ±0.025697 | 0.291  ±0.017059 | 0.205333  ±0.007506 |
| 0.75 M | 0.069  ±0.009644 | 0.099333  ±0.004041 | 0.149667  ±0.016258 | 0.171  ±0.026287 | 0.164333  ±0.022811 | 0.157667  ±0.012097 |
| 1 M | 0.070333  ±0.023544 | 0.086333  ±0.004041 | 0.13  ±0.024249 | 0.131667  ±0.009074 | 0.125333  ±0.018583 | 0.122667  ±0.016653 |
| Note: Values are means of three biological replicates ± standard deviation. | | | | | | |

Table S2. **Measurement of MDA content in *P. indica* treated with** different salt (NaCl) concentrations.

| NaCl | Total TBARS (nmole/g FW) | **Standard deviation (**±) |
| --- | --- | --- |
| **0M** | 3.6425 | 0.246086 |
| **0.5 M** | 5.3525 | 0.832721 |
| **1 M** | 8.0925 | 0.819771 |
| Note: MDA content was measured in terms of total TBARS (thiobarbituric acid reactive substance).Values are means of at least four biological replicates ± standard deviation. | | |

Table S3. EuKaryotic Orthologous Groups (KOG) class enrichment for DEGs in *P. indica* **after treatment with 0.5M NaCl for 14 days.**

| **Group** | **Term** | **nseqs** | **delta.rank** | **pval** | **padj** |
| --- | --- | --- | --- | --- | --- |
| 1 | Translation, ribosomal structure and biogenesis | 29 | -82 | 0.00001 | 0.00024 |
| 14 | Cytoskeleton | 27 | -78 | 0.00004 | 0.00050 |
| 9 | Amino acid transport and metabolism | 20 | 54 | 0.01459 | 0.12161 |
| 22 | Chromatin structure and dynamics | 6 | 89 | 0.02330 | 0.14565 |
| 24 | Replication, recombination and repair | 6 | 79 | 0.04529 | 0.22646 |
| 20 | Nucleotide transport and metabolism | 4 | 81 | 0.09096 | 0.32753 |
| 11 | Energy production and conversion | 14 | 42 | 0.10429 | 0.32753 |
| 18 | Intracellular trafficking, secretion, and vesicular transport | 6 | 61 | 0.11980 | 0.32753 |
| 25 | Coenzyme transport and metabolism | 1 | 146 | 0.12539 | 0.32753 |
| 16 | Inorganic ion transport and metabolism | 6 | 59 | 0.13101 | 0.32753 |
| 15 | Transcription | 11 | -40 | 0.16894 | 0.38396 |
| 13 | Lipid transport and metabolism | 27 | 22 | 0.25441 | 0.41340 |
| 6 | General function prediction only | 39 | 18 | 0.26226 | 0.41340 |
| 3 | Carbohydrate transport and metabolism | 26 | 22 | 0.26836 | 0.41340 |
| 19 | Cell wall/membrane/envelope biogenesis | 8 | -38 | 0.27087 | 0.41340 |
| 7 | Function unknown | 30 | -19 | 0.29262 | 0.41340 |
| 2 | Cell cycle control, cell division, chromosome partitioning | 5 | -45 | 0.29710 | 0.41340 |
| 17 | RNA processing and modification | 6 | 40 | 0.30648 | 0.41340 |
| 10 | Extracellular structures | 10 | -31 | 0.31418 | 0.41340 |
| 21 | Nuclear structure | 2 | 57 | 0.40347 | 0.50434 |
| 8 | Posttranslational modification, protein turnover, chaperones | 15 | 19 | 0.45464 | 0.54124 |
| 23 | no description | 1 | 63 | 0.51373 | 0.57780 |
| 5 | Signal transduction mechanisms | 9 | -20 | 0.53157 | 0.57780 |
| 12 | Defense mechanisms | 1 | 35 | 0.72025 | 0.75027 |
| 4 | Secondary metabolites biosynthesis, transport and catabolism | 20 | -4 | 0.84038 | 0.84038 |
| Note: Delta.rank represents the differences between mean rank of genes belonging to a particular KOG class and mean rank of all other genes. Singificance level padj<0.01. | | | | | |
|  |  |  |  |  |  |

**Table S4. List of DEGs related to transporter proteins and transcription factors in *P. indica* treated with 0.5M NaCl for 14 days.**

| **Unigene ID** | **Gene ID** | **Log2 fold change** | | **Description** | **KOG Defline** |
| --- | --- | --- | --- | --- | --- |
| **Transporter proteins** | | | | | |
| unigene_11470 | gene_9866 | 2.8 | | Predicted transporter (major facilitator superfamily) | Predicted transporter (major facilitator superfamily) |
| unigene_12177 | gene_7707 | -1.9 | | probable DHA14-like major facilitator; ABC transporter | Predicted transporter (major facilitator superfamily) |
| unigene_12332 | gene_8187 | 2.9 | | phosphate transporter, probable PHO84-Inorganic phosphate permease | Inorganic phosphate transporter |
| unigene_12422 | gene_6083 | -2.4 | | probable DHA14-like major facilitator; ABC transporter [ | Predicted transporter (major facilitator superfamily) |
| unigene_13131 | gene_8171 | 2.3 | | related to major facilitator MirA | Predicted transporter (major facilitator superfamily) |
| unigene_13229 | gene_8170 | 3.3 | | related to major facilitator MirA | Predicted transporter (major facilitator superfamily) |
| unigene_2097 | gene_6696 | -4.1 | | related to putative tartrate transporter | Permease of the major facilitator superfamily |
| unigene_4520 | gene_6083 | -2.8 | | probable DHA14-like major facilitator; ABC transporter | Predicted transporter (major facilitator superfamily) |
| unigene_6533 | gene_2173 | -3.5 | | probable high-affinity glucose | Predicted transporter (major facilitator superfamily) |
| unigene_3386 | gene_5072 | -3.2 | | Ca2+-modulated nonselective cation channel polycystin | Ca2+-modulated nonselective cation channel polycystin |
| unigene_4289 | gene_5070 | -2.4 | | Ca2+-modulated nonselective cation channel polycystin | Ca2+-modulated nonselective cation channel polycystin |
| unigene_3627 | gene_3033 | -3.1 | | related to PTR2-Di-and tripeptide | H+/oligopeptide symporter |
| unigene_5422 | gene_607 | 1.9 | | hypothetical protein PIIN_00596 | Predicted divalent cation transporter |
| unigene_11316 | gene_3446 | -2.0 | | hypothetical protein PIIN_03395 | Voltage-gated shaker-like K+ channel, subunit beta/KCNAB |
| unigene_11780 | gene_1075 | 2.6 | | related to aryl-alcohol dehydrogenases | Voltage-gated shaker-like K+ channel, subunit beta/KCNAB |
| unigene_11781 | gene_1075 | 2.6 | | related to aryl-alcohol dehydrogenases | Voltage-gated shaker-like K+ channel, subunit beta/KCNAB |
| unigene_11782 | gene_1075 | 3.6 | | related to aryl-alcohol dehydrogenases | Voltage-gated shaker-like K+ channel, subunit beta/KCNAB |
| unigene_13567 | gene_1883 | 2.5 | | Calcium-transporting ATPase. | Ca2+ transporting ATPase |
| **Transcription factors** | | | | |  |
| unigene_1770 | gene_7480 | 3.0 | hypothetical protein PIIN_07425 | | Putative transcription factor 5qNCA, contains JmjC domain |
| unigene_3444 | gene_4361 | -2.2 | related to n-alkane-inducible cytochrome P450 | | Transcription factor, contains HOX domain |
| unigene_3710 | gene_444 | -2.9 | hypothetical protein PIIN_00439 | | HMG-box transcription factor |
| unigene_4954 | gene_8305 | -2.4 | hypothetical protein PIIN_08249 | | Putative transcription factor HALR/MLL3, involved in embryonic development |
| unigene_8170 | gene_1409 | -6.9 | related to putative phosphatidylserine decarboxylase-Burkholderia xenovorans | | Helicase-like transcription factor HLTF/DNA helicase RAD5, DEAD-box superfamily |

**Table S5. List of salt-responsive differentially expressed genes in *P. indica* after treatment with 0.5M NaCl for 14 days.**

| **Unigene ID** | ***P. indica* Gene ID** | **Description** | **Log2 Fold Change** | **KOG Defline** |
| --- | --- | --- | --- | --- |
| **DEGs involved in Cell Wall biogenesis/modification** | | | | |
| unigene_14619 | gene_4567 | related to proteophosphoglycan ppg4 | -2.9 | Chitinase |
| unigene_5432 | gene_3274 | hypothetical protein PIIN_03222 | -2.2 | Chitinase |
| unigene_5652 | gene_6314 | hypothetical protein PIIN_06258 | -6.8 | Chitinase |
| unigene_5860 | gene_2013 | related to CDA2-sporulation-specific chitin deacetylase | -4.1 | - |
| unigene_7100 | gene_3594 | probable endochitinase | -3.0 | Chitinase |
| unigene_8866 | gene_8785 | hypothetical protein PIIN_08725 | 4.6 | Chitinase |
| unigene_9741 | gene_3594 | probable endochitinase | -2.7 | Chitinase |
| unigene_3157 | gene_9281 | related to endo-1,3(4)-beta-glucanase | -7.0 | - |
| unigene_4931 | gene_491 | related to beta-1,6-glucanase precursor | -2.0 | Beta-glucocerebrosidase |
| unigene_10867 | gene_1581 | related to beta-glucosidase | 3.2 | - |
| unigene_12374 | gene_5553 | probable mutanase (glucan endo-1,3-alpha-glucosidase | 2.2 | beta-1,6-N-acetylglucosaminyltransferase, contains WSC domain |
| unigene_3291 | gene_1536 | probable glucan 1,3-beta-glucosidase | -6.4 | beta-1,6-N-acetylglucosaminyltransferase, contains WSC domain |
| unigene_5319 | gene_5776 | related to alpha-glucosidase b | -1.9 | Maltase glucoamylase and related hydrolases, glycosyl hydrolase family 31 |
| unigene_5991 | gene_7471 | related to beta-glucosidase | -2.5 | - |
| unigene_6414 | gene_4770 | hypothetical protein PIIN_04715 | -1.7 | Unnamed protein |
| unigene_1792 | gene_9654 | related to Rho3 GTP binding protein | -4.3 | Ras-related small GTPase, Rho type |
| unigene_9456 | gene_10432 | probable GTPase Rho1 | 2.9 | Ras-related small GTPase, Rho type |
| unigene_10360 | gene_9741 | glycoside hydrolase family 16 protein | 3.7 |  |
| unigene_12108 | gene_4489 | hypothetical protein PIIN_04436 | 2.3 | Predicted glycosyltransferase |
| unigene_14604 | gene_9926 | hypothetical protein M408DRAFT_329690 | -2.1 | GPI transamidase complex, GPI17/PIG-S component, involved in glycosylphosphatidylinositol anchor biosynthesis |
| **DEGs involved in Oxidative stress** | | | | |
| unigene_11989 | gene_3137 | related to glutathione synthase | -1.9 | Glutathione synthetase |
| unigene_3628 | gene_3137 | related to glutathione synthase | -2.2 | Glutathione synthetase |
| unigene_5009 | gene_1643 | related to URE2-nitrogen catabolite repression regulator | -3.2 | Glutathione S-transferase |
| unigene_2182 | gene_2714 | probable thioredoxin peroxidase | 2.6 | Alkyl hydroperoxide reductase, thiol specific antioxidant and related enzymes |
| unigene_3147 | gene_1820 | related to thioredoxin | -2.4 | Thioredoxin |
| unigene_3148 | gene_1820 | related to thioredoxin | -2.2 | Thioredoxin |
| unigene_8122 | gene_6511 | related to FAP7-involved in the oxidative stress response | 2.1 | Predicted nucleotide kinase/nuclear protein involved oxidative stress response |
| unigene_11233 | gene_762 | hypothetical protein PIIN_00746 | -2.9 | Zinc-binding oxidoreductase |
| unigene_9704 | gene_8668 | hypothetical protein PIIN_08610 | 4.8 | Ubiquinol-cytochrome c reductase hinge protein |
| unigene_7004 | - | cytochrome c oxidase subunit III (mitochondrion) | -6.6 | - |
| unigene_2387 | - | cytochrome c oxidase subunit I (mitochondrion) | -6.7 | - |
| unigene_489 | - | CYTB gene product (mitochondrion) | -5.7 | - |
| unigene_2057 | gene_3870 | hypothetical protein PIIN_03819 | -2.5 | Kynurenine 3-monooxygenase and related flavoprotein monooxygenases |
| unigene_5372 | gene_4793 | related to monooxygenase | 2.2 | Flavin-containing monooxygenase |
| unigene_12730 | gene_4793 | related to monooxygenase | 2.2 | Flavin-containing monooxygenase |
| unigene_12731 | gene_4793 | related to monooxygenase | 2.3 | Flavin-containing monooxygenase |
| unigene_2057 | gene_3870 | hypothetical protein PIIN_03819 | -2.5 | Kynurenine 3-monooxygenase and related flavoprotein monooxygenases |
| unigene_4566 | gene_6428 | related to tyrosinase precursor (monophenol monooxygenase) | -2.8 | - |
| unigene_13081 | gene_8060 | related to glutamate decarboxylase | 2.0 | Glutamate decarboxylase/sphingosine phosphate lyase |
| unigene_3774 | gene_8060 | related to glutamate decarboxylase | 2.6 | Glutamate decarboxylase/sphingosine phosphate lyase |
| **DEGs related to sugar and polyamines** | | | | |
| unigene_4374 | gene_4049 | related to formaldehyde dehydrogenase | 2.7 | Sorbitol dehydrogenase |
| unigene_4375 | gene_4049 | related to formaldehyde dehydrogenase | 2.7 | Sorbitol dehydrogenase |
| unigene_11518 | gene_3488 | probable chimeric spermidine synthase/saccharopine reductase | 2.0 | Lysine-ketoglutarate reductase/saccharopine dehydrogenase |
| **DEGs involved in Sterol biosynthesis** | | | | |
| unigene_10637 | gene_9787 | related to ERG27-3-keto sterol reductase | 2.7 | 3-keto sterol reductase |
| unigene_13355 | gene_5871 | probable hydroxymethylglutaryl-CoA synthase | 3.0 | Hydroxymethylglutaryl-CoA synthase |
| unigene_3108 | gene_8416 | probable ERG5-C-22 sterol desaturase | 2.7 | Cytochrome P450 CYP4/CYP19/CYP26 subfamilies |
| unigene_8750 | gene_8297 | probable delta(24)-sterol c-methyltransferase (erg6) | 2.2 | SAM-dependent methyltransferases |
| unigene_13794 | gene_407 | Lanosterol synthase.Terpene cyclase/mutase family member | 2.5 | Oxidosqualene-lanosterol cyclase and related proteins |
| unigene_8199 | gene_2824 | probable stearoyl-CoA desaturase | 2.4 | Fatty acid desaturase |
| **DEGs involved in Signal transduction** | | | | |
| unigene_12963 | gene_3048 | hypothetical protein PIIN_02997 (Mitogen-activated protein kinase) | -1.8 | - |
| unigene_307 | gene_3712 | probable casein kinase-1 hhp1 | -5.5 | Casein kinase (serine/threonine/tyrosine protein kinase) |
| unigene_5572 | gene_7983 | hypothetical protein PIIN_07928 | -1.8 | Serine/threonine protein kinase |
| unigene_8491 | gene_6302 | hypothetical protein PIIN_06246 | -4.8 | Cdc2-related protein kinase |
| unigene_11075 | gene_1199 | hypothetical protein PIIN_01174 | -3.1 | C-type lectin |
| unigene_11389 | gene_3590 | hypothetical protein PIIN_03539 | 1.9 | Nucleolar GTPase/ATPase p130 |
| unigene_13189 | gene_1611 | related to guanine nucleotide-binding protein alpha-2 subunit | -2.0 | G protein subunit Galphaq/Galphay, small G protein superfamily |
| unigene_1600 | gene_6968 | hypothetical protein PIIN_06914 | -3.5 | FOG: Hormone receptors |
| unigene_2128 | gene_5751 | hypothetical protein PIIN_05696 | -2.8 | C-type lectin |
| unigene_2281 | gene_3047 | hypothetical protein PIIN_02996 | -2.0 | Dual specificity phosphatase |
| unigene_3471 | gene_2436 | related to beta-1,3-glucan binding protein | 2.4 | Microtubule-associated serine/threonine kinase and related proteins |
| unigene_4513 | gene_1611 | related to guanine nucleotide-binding protein alpha-2 subunit | -2.0 | G protein subunit Galphaq/Galphay, small G protein superfamily |
| unigene_5205 | gene_1275 | hypothetical protein PIIN_01251 | -2.0 | Nucleolar GTPase/ATPase p130 |
| unigene_5572 | gene_7983 | hypothetical protein PIIN_07928 | -1.8 | Serine/threonine protein kinase |
| unigene_10289 | gene_556 | hypothetical protein PIIN_00547 | 4.7 | protein kinase activity [GO:0004672] |
| unigene_4954 | gene_8305 | hypothetical protein PIIN_08249 | -2.4 | - |
| unigene_1792 | gene_9654 | related to Rho3 GTP binding protein | -4.3 | Ras-related small GTPase, Rho type |
| unigene_9456 | gene_10432 | probable GTPase Rho1 | 2.9 | Ras-related small GTPase, Rho type |
| unigene_14604 | gene_9926 | hypothetical protein M408DRAFT_329690 | -2.1 | GPI transamidase complex, GPI17/PIG-S component, involved in glycosylphosphatidylinositol anchor biosynthesis |
| unigene_2611 | gene_1619 | related to Type 2C Protein Phosphatase | -3.2 | Protein phosphatase 2C/pyruvate dehydrogenase (lipoamide) phosphatase |
| unigene_4146 | gene_2489 | hypothetical protein PIIN_02443 (Diacylglycerol kinase (ATP)) | 2.2 | - |
| unigene_11878 | gene_2489 | hypothetical protein PIIN_02443 (Diacylglycerol kinase (ATP)) | 2.2 | - |
| **DEGs related to Cytochrome P450** | | | | |
| unigene_11721 | gene_10490 | related to cytochrome P450-Trametes versicolor | 2.4 | Cytochrome P450 CYP3/CYP5/CYP6/CYP9 subfamilies |
| unigene_11800 | gene_4361 | related to n-alkane-inducible cytochrome P450 | -2.5 | Cytochrome P450 CYP4/CYP19/CYP26 subfamilies |
| unigene_11801 | gene_4361 | related to n-alkane-inducible cytochrome P450 | -2.4 | Cytochrome P450 CYP4/CYP19/CYP26 subfamilies |
| unigene_12293 | gene_10583 | probable cycloheximide-inducible protein CIP70 (cytochrome P450 family) | 2.2 | Cytochrome P450 CYP4/CYP19/CYP26 subfamilies |
| unigene_2276 | gene_5471 | hypothetical protein PIIN_05418 | -4.5 | Cytochrome P450 CYP2 subfamily |
| unigene_301 | gene_5469 | related to O-methylsterigmatocystin oxidoreductase | -3.7 | Cytochrome P450 CYP2 subfamily |
| unigene_3081 | gene_5471 | hypothetical protein PIIN_05418 | -2.0 | Cytochrome P450 CYP2 subfamily |
| unigene_3207 | gene_9502 | related to Cytochrome P450 | -1.9 | Cytochrome P450 CYP4/CYP19/CYP26 subfamilies |
| unigene_3443 | gene_4361 | related to n-alkane-inducible cytochrome P450 | -2.6 | Cytochrome P450 CYP4/CYP19/CYP26 subfamilies |
| unigene_3444 | gene_4361 | related to n-alkane-inducible cytochrome P450 | -2.2 | Cytochrome P450 CYP4/CYP19/CYP26 subfamilies |
| unigene_3445 | gene_4361 | related to n-alkane-inducible cytochrome P450 | -2.6 | Cytochrome P450 CYP4/CYP19/CYP26 subfamilies |
| unigene_3446 | gene_4361 | related to n-alkane-inducible cytochrome P450 | -2.2 | Cytochrome P450 CYP4/CYP19/CYP26 subfamilies |
| unigene_3447 | gene_4361 | related to n-alkane-inducible cytochrome P450 | -2.6 | Cytochrome P450 CYP4/CYP19/CYP26 subfamilies |
| **DEGs involved in Chromatin structure and dynamics** | | | | |
| unigene_6305 | gene_2526 | hypothetical protein PIIN_02481 | 2.2 | Histone H3 (Lys9) methyltransferase SUV39H1/Clr4, required for transcriptional silencing |
| unigene_6308 | gene_2526 | hypothetical protein PIIN_02481 | 2.1 | Histone H3 (Lys9) methyltransferase SUV39H1/Clr4, required for transcriptional silencing |
| unigene_6310 | gene_2526 | hypothetical protein PIIN_02481 | 2.0 | Histone H3 (Lys9) methyltransferase SUV39H1/Clr4, required for transcriptional silencing |
| unigene_14250 | gene_2526 | Related to dihydroorotate dehydrogenase a | 1.8 | Histone H3 (Lys9) methyltransferase SUV39H1/Clr4, required for transcriptional silencing |
| unigene_8239 | gene_1663 | hypothetical protein PIIN_11732 | 2.9 | Predicted histone tail methylase containing SET domain |
| unigene_8320 | gene_7378 | hypothetical protein PIIN_07324 | -2.0 | SWI-SNF chromatin-remodeling complex protein |
| unigene_13221 | gene_7552 | related to meiotic recombination protein rec8 | 2.8 | Sister chromatid cohesion complex Cohesin, subunit RAD21/SCC1 |
| unigene_3299 | gene_6967 | related to GLE2-required for nuclear pore complex structure and function | -2.0 | Mitotic spindle checkpoint protein BUB3, WD repeat superfamily |
| unigene_166 | gene_7963 | PREDICTED: translationally-controlled tumor protein isoform X1 | -5.8 | Microtubule-binding protein (translationally controlled tumor protein) |
| **DEGs related to Proteases** | | | | |
| unigene_11216 | gene_7611 | related to metalloprotease MEP1 | -2.4 | - |
| unigene_13173 | gene_6402 | hypothetical protein PIIN_06346, partial | 2.6 | Aspartyl protease |
| unigene_3389 | gene_6360 | related to deuterolysin M35 metalloprotease | -3.4 | - |
| unigene_5265 | gene_6360 | related to deuterolysin M35 metalloprotease | -2.9 | - |
| unigene_5266 | gene_6360 | related to deuterolysin M35 metalloprotease | -3.0 | - |
| unigene_5267 | gene_6360 | related to deuterolysin M35 metalloprotease | -3.1 | - |
| unigene_9129 | gene_2131 | hypothetical protein PIIN_02088 | 2.3 | Serine proteinase inhibitor (KU family) with thrombospondin repeats |
| unigene_14629 | gene_9112 | probable extracellular elastinolytic metalloproteinase precursor | -2.3 | - |
| unigene_6266 | gene_429 | probable extracellular elastinolytic metalloproteinase precursor | -1.8 | beta-1,6-N-acetylglucosaminyltransferase, contains WSC domain |
| unigene_7302 | gene_9112 | probable extracellular elastinolytic metalloproteinase precursor | -3.9 | - |
| **DEGs related to WD40 repeat protein** | | | | |
| unigene_12344 | gene_5700 | hypothetical protein PIIN_05645 | -2.2 | Uncharacterized conserved protein, contains WD40 repeats |
| unigene_13510 | gene_5401 | Related to WD40-repeat protein (Notchless protein) | 2.8 | FOG: WD40 repeat |
| unigene_13516 | gene_10511 | Uncharacterized protein | 2.5 | U4/U6 small nuclear ribonucleoprotein Prp4 (contains WD40 repeats) |
| unigene_13517 | gene_2964 | Related to WD40-repeat protein (Notchless protein) | 2.6 | WD40 repeat-containing protein |
| unigene_13722 | gene_5401 | Related to WD40-repeat protein (Notchless protein) | 3.8 | FOG: WD40 repeat |
| unigene_1448 | gene_7521 | related to WD40-repeat protein (notchless protein) | -4.8 | Notchless-like WD40 repeat-containing protein |
| unigene_1449 | gene_7521 | related to WD40-repeat protein (notchless protein) | -4.6 | Notchless-like WD40 repeat-containing protein |
| unigene_14647 | gene_7521 | related to WD40-repeat protein (notchless protein) | -3.8 | Notchless-like WD40 repeat-containing protein |
| unigene_14652 | gene_4362 | hypothetical protein PIIN_04311 | -3.7 | Notchless-like WD40 repeat-containing protein |
| unigene_14681 | gene_7521 | related to WD40-repeat protein (notchless protein) | -4.5 | Notchless-like WD40 repeat-containing protein |
| unigene_14736 | gene_7521 | related to WD40-repeat protein (notchless protein) | -4.4 | Notchless-like WD40 repeat-containing protein |
| unigene_3822 | gene_3608 | related to RRP9-protein associated with the U3 small nucleolar RNA | -1.8 | U3 snoRNP-associated protein (contains WD40 repeats) |
| unigene_3824 | gene_3608 | related to RRP9-protein associated with the U3 small nucleolar RNA | -2.2 | U3 snoRNP-associated protein (contains WD40 repeats) |
| unigene_466 | gene_8493 | hypothetical protein PIIN_08437 | -3.0 | Notchless-like WD40 repeat-containing protein |
| unigene_467 | gene_8493 | hypothetical protein PIIN_08437 | -2.9 | Notchless-like WD40 repeat-containing protein |
| unigene_4688 | gene_10326 | hypothetical protein PIIN_10254 | -2.4 | WD40-repeat-containing subunit of the 18S rRNA processing complex |
| unigene_5620 | gene_670 | related to WD40-repeat protein (notchless protein) | -2.6 | Notchless-like WD40 repeat-containing protein |
| unigene_5620 | gene_670 | related to WD40-repeat protein (notchless protein) | -2.6 | Notchless-like WD40 repeat-containing protein |
| unigene_5779 | gene_9957 | hypothetical protein PIIN_09893 | -3.0 | Notchless-like WD40 repeat-containing protein |
| unigene_5945 | gene_9918 | related to WD40-repeat protein (notchless protein) | -3.0 | Notchless-like WD40 repeat-containing protein |
| unigene_5946 | gene_9918 | related to WD40-repeat protein (notchless protein) | -3.6 | Notchless-like WD40 repeat-containing protein |
| unigene_6757 | gene_4909 | related to WD40-repeat protein (notchless protein) | -4.0 | Notchless-like WD40 repeat-containing protein |
| unigene_9688 | gene_11638 | hypothetical protein PIIN_11541, partial | 2.8 | WD40 repeat-containing protein |
| unigene_9703 | gene_8355 | WD40 repeat | 4.0 | Notchless-like WD40 repeat-containing protein |

Table S6. **Unigenes used for validation of gene expression profile by RT-qPCR analysis** **in *P. indica* transcriptome data.**

| **Unigene ID** | **Avg RQ (2^-⊿⊿ Ct^)** | **Std** | **Log2 fold change** | **RNA-Seq** |
| --- | --- | --- | --- | --- |
| unigene_10060 | 4.897382 | ±0.211655 | 2.292011 | 3.5 |
| unigene_10360 | 2.14051 | ±0.045748 | 1.097954 | 3.7 |
| unigene_10463 | 0.433323 | ±0.036782 | -1.20648 | -2.3 |
| unigene_10867 | 1.184362 | ±0.111745 | 0.24411 | 3.2 |
| unigene_10899 | 1.700138 | ±0.084617 | 0.765652 | 2.2 |
| unigene_1117 | 0.488239 | ±0.024402 | -1.03434 | -2.2 |
| unigene_9741 | 0.56628 | ±0.03555 | -0.82041 | -2.7 |
| unigene_8403 | 2.758165 | ±0.081778 | 1.463709 | 3.3 |
| unigene_6533 | 0.601124 | ±0.031193 | -0.73427 | -3.5 |
| unigene_11359 | 5.604883 | ±0.757174 | 2.486684 | 2.9 |
| unigene_12332 | 3.160603 | ±0.379959 | 1.6602 | 2.9 |
| unigene_12422 | 0.972137 | ±0.055159 | -0.04077 | -2.4 |
| unigene_13081 | 4.448417 | ±0.304268 | 2.153292 | 2 |
| Note: Relative gene expression level (2^-⊿⊿ Ct^) with *PiTEF* used as the reference gene in RT-qPCR analysis. Data are presented as means ± standard deviation (SD) of three replicates. | | | | |

Table S7. Primers used in **RT-qPCR analysis** for validation of gene expression profile in P. indica transcriptome data.

| Unigene ID | **Description** | Primer sequences | Amplified products |
| --- | --- | --- | --- |
| unigene_10060 | probable UDP-glucose 6-dehydrogenase | Forward: TCCAAGAACCGCAATCCTCT | 138 bp |
|  |  | Reverse: TCGTTGCATCTCCCAGAAGT |  |
| unigene_10360 | glycoside hydrolase family 16 protein | Forward: CGAAATGGTGCTGGTGTTCA | 115 bp |
|  |  | Reverse: GCTAACATGCGGAGAAGACG |  |
| unigene_10463 | probable 6-phosphofructokinase | Forward: CCCCAATCGTCAAATCGGTC | 110 bp |
|  |  | Reverse: CAAAGGATCCATCACTGCCG |  |
| unigene_10867 | related to beta-glucosidase | Forward: CGAGCCAAAGTTCCAAAGCT | 134 bp |
|  |  | Reverse: TCACTCAGCGTATCGACTCC |  |
| unigene_10899 | related to alpha-isopropylmalate isomerase | Forward: TCATGAGCAAGGACGATGGT | 144 bp |
|  |  | Reverse: CCGCCTTTCCAATAGCAGAC |  |
| unigene_1117 | related to CYP-41 peptidyl-prolyl cis-trans isomerase (cyclophilin41) | Forward: ATCTCCCGCTGTAGTCTTGG | 114 bp |
|  |  | Reverse: ATCACGACCGTTCCTACTCC |  |
| unigene_9741 | probable endochitinase | Forward: TACACAGCGGGGATTCAAGT | 138 bp |
|  |  | Reverse: GCCTTCATACACTCTTGGCG |  |
| unigene_8403 | probable GTPase Rho1 | Forward: CGCAGCTCTTCGACTTCTTC | 121 bp |
|  |  | Reverse: GCTTGCCCTTTGATACAGCA |  |
| unigene_6533 | probable high-affinity glucose transporter | Forward: TCCCGTGATTACCCAGACAC | 142 bp |
|  |  | Reverse: CATCGATAAATGGGGCCGTC |  |
| unigene_11359 | related to SLC1-1-acyl-sn-gylcerol-3-phosphate acyltransferase | Forward: TCGACCGTTCATCCTCTCAG | 124 bp |
|  |  | Reverse: TCCTTGCTGAGTGTCCTTGT |  |
| unigene_12332 | phosphate transporter | Forward: AGCTGCCTTCATCAAGTCCT | 116 bp |
|  |  | Reverse: TCTTCCTCGACGCCTATGAC |  |
| unigene_12422 | probable DHA14-like major facilitator; ABC transporter | Forward: TTGGTTTCGGTGTAGGCTCT | 119 bp |
|  |  | Reverse: GACGAGCTGGGCAAAACTAG |  |
| unigene_13081 | related to glutamate decarboxylase | Forward: CGGCCTTGGGTGCATTATAG | 115 bp |
|  |  | Reverse: TTCCGCCTCTCAGACGAATT |  |
| AJ249912.1 | Translation elongation factor (tef) | Forward: TCGTCGCTGTCAACAAGATG | 163bp |
|  |  | Reverse: GAGGGCTCGAGCATGTTGT |  |
