# Supplementary material for: A high-throughput RNA-Seq approach to elucidate the transcriptional response of Piriformospora indica to high salt stress
Source: Sci Rep. 2021 Feb 18;11:4129. doi: 10.1038/s41598-021-82136-0 (PMC7893156; doi:10.1038/s41598-021-82136-0)

Additional supplementary information:

**Title: A high-throughput RNA-Seq approach to elucidate the transcriptional response of *Piriformospora indica* to high salt stress.**

**Authors: Nivedita^1^, Abdul Rawoof^2^, Nirala Ramchiary^2^, *Malik Z. Abdin^1^.**

**Affiliation:** 1. Department of Biotechnology, Jamia Hamdard, New Delhi, India.

2. School of Life sciences, Jawaharlal Nehru University, New Delhi, India.

*Corresponding author:

*Malik Z. Abdin- Email: mzabdin@jamiahamdard.ac.in,

Ph: +919818462060

**Table S8. Statistical analysis of *P. indica* dry weight measurements treated with different salt (NaCl) concentrations over different time intervals.**

**One-way Anova analysis:**

| **Descriptives** | | | | | | | | | | | | | | | | | | | | |
| --- | --- | --- | --- | --- | --- | --- | --- | --- | --- | --- | --- | --- | --- | --- | --- | --- | --- | --- | --- | --- |
| DW | |  | | |  | | |  | | |  | | |  | |  | | |  |  |
|  | | N | | | Mean | | | Std. Deviation | | | Std. Error | | | 95% Confidence Interval for Mean | | | | | Minimum | Maximum |
|  |  |  |  |  |  |  |  |  |  |  |  |  |  | Lower Bound | | Upper Bound | | |  |  |
| 0M | | 6 | | | .3567 | | | .17829 | | | .07279 | | | .1696 | | .5438 | | | .07 | .54 |
| 0.25M | | 6 | | | .2733 | | | .12691 | | | .05181 | | | .1401 | | .4065 | | | .07 | .39 |
| 0.5M | | 6 | | | .1983 | | | .08954 | | | .03655 | | | .1044 | | .2923 | | | .07 | .30 |
| 0.75M | | 6 | | | .1350 | | | .04037 | | | .01648 | | | .0926 | | .1774 | | | .07 | .17 |
| 1M | | 6 | | | .1117 | | | .02563 | | | .01046 | | | .0848 | | .1386 | | | .07 | .13 |
| Total | | 30 | | | .2150 | | | .13594 | | | .02482 | | | .1642 | | .2658 | | | .07 | .54 |
|  | |  | | |  | | |  | | |  | | |  | |  | | |  |  |
|  | |  | | |  | | |  | | |  | | |  | |  | | |  |  |
| **Test of Homogeneity of Variances** | | | | | | | | | | |  | | |  | |  | | |  |  |
| DW | |  | | |  | | |  | | |  | | |  | |  | | |  |  |
| Levene Statistic | | df1 | | | df2 | | | Sig. | | |  | | |  | |  | | |  |  |
| 5.133 | | 4 | | | 25 | | | .004 | | |  | | |  | |  | | |  |  |
| **ANOVA** | | | | | | | | | | | | | | | | | |  |  |  |
| DW |  | | |  | | |  | | |  | | |  | | | | |  |  |  |
|  | Sum of Squares | | | df | | | Mean Square | | | F | | | Sig. | | | | |  |  |  |
| Between Groups | .245 | | | 4 | | | .061 | | | 5.262 | | | .003 | | | | |  |  |  |
| Within Groups | .291 | | | 25 | | | .012 | | |  | | |  | | | | |  |  |  |
| Total | .536 | | | 29 | | |  | | |  | | |  | | | | |  |  |  |
| **Multiple Comparisons** | | | | | | | | | | | | | | | | | |  |  |  |
| Dependent Variable: | | | DW | | |  | | |  | | |  | | |  | |  |  |  |  |
| Tukey HSD | | |  | | |  | | |  | | |  | | |  | |  |  |  |  |
| (I) NaCl | | | | | | Mean Difference (I-J) | | | Std. Error | | | Sig. | | | 95% Confidence Interval | | |  |  |  |
|  |  |  |  |  |  |  |  |  |  |  |  |  |  |  | Lower Bound | | Upper Bound |  |  |  |
| 0M | | | 0.25M | | | .08333 | | | .06229 | | | .671 | | | -.0996 | | .2663 |  |  |  |
|  |  |  | 0.5M | | | .15833 | | | .06229 | | | .113 | | | -.0246 | | .3413 |  |  |  |
|  |  |  | 0.75M | | | .22167^*^ | | | .06229 | | | .012 | | | .0387 | | .4046 |  |  |  |
|  |  |  | 1M | | | .24500^*^ | | | .06229 | | | .005 | | | .0621 | | .4279 |  |  |  |
| 0.25M | | | 0M | | | -.08333 | | | .06229 | | | .671 | | | -.2663 | | .0996 |  |  |  |
|  |  |  | 0.5M | | | .07500 | | | .06229 | | | .749 | | | -.1079 | | .2579 |  |  |  |
|  |  |  | 0.75M | | | .13833 | | | .06229 | | | .205 | | | -.0446 | | .3213 |  |  |  |
|  |  |  | 1M | | | .16167 | | | .06229 | | | .102 | | | -.0213 | | .3446 |  |  |  |
| 0.5M | | | 0M | | | -.15833 | | | .06229 | | | .113 | | | -.3413 | | .0246 |  |  |  |
|  |  |  | 0.25M | | | -.07500 | | | .06229 | | | .749 | | | -.2579 | | .1079 |  |  |  |
|  |  |  | 0.75M | | | .06333 | | | .06229 | | | .845 | | | -.1196 | | .2463 |  |  |  |
|  |  |  | 1M | | | .08667 | | | .06229 | | | .639 | | | -.0963 | | .2696 |  |  |  |
| 0.75M | | | 0M | | | -.22167^*^ | | | .06229 | | | .012 | | | -.4046 | | -.0387 |  |  |  |
|  |  |  | 0.25M | | | -.13833 | | | .06229 | | | .205 | | | -.3213 | | .0446 |  |  |  |
|  |  |  | 0.5M | | | -.06333 | | | .06229 | | | .845 | | | -.2463 | | .1196 |  |  |  |
|  |  |  | 1M | | | .02333 | | | .06229 | | | .996 | | | -.1596 | | .2063 |  |  |  |
| 1M | | | 0M | | | -.24500^*^ | | | .06229 | | | .005 | | | -.4279 | | -.0621 |  |  |  |
|  |  |  | 0.25M | | | -.16167 | | | .06229 | | | .102 | | | -.3446 | | .0213 |  |  |  |
|  |  |  | 0.5M | | | -.08667 | | | .06229 | | | .639 | | | -.2696 | | .0963 |  |  |  |
|  |  |  | 0.75M | | | -.02333 | | | .06229 | | | .996 | | | -.2063 | | .1596 |  |  |  |
| *. The mean difference is significant at the 0.05 level. | | | | | | | | | | | | | | | | | |  |  |  |

**Means Plots:**


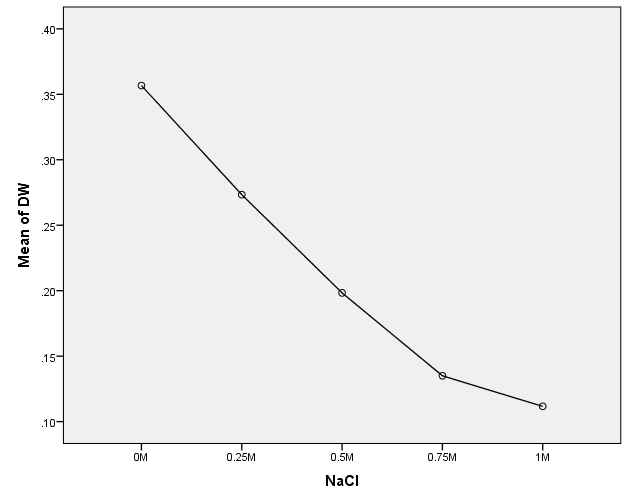


**Table S9. Statistical analysis of MDA content measurements in *P. indica* treated with different salt (NaCl) concentrations.**

**One-way Anova analysis:**

| **Descriptives (MDA content)** | | | | | | | | |
| --- | --- | --- | --- | --- | --- | --- | --- | --- |
|  |  |  |  |  |  |  |  |  |
| NaCl | N | Mean | Std. Deviation | Std. Error | 95% Confidence Interval for Mean | | Minimum | Maximum |
|  |  |  |  |  | Lower Bound | Upper Bound |  |  |
| Control (0M) | 4 | 3.6425 | .24609 | .12304 | 3.2509 | 4.0341 | 3.48 | 4.00 |
| salt1 (0.5M) | 4 | 5.3525 | .83272 | .41636 | 4.0275 | 6.6775 | 4.38 | 6.32 |
| salt2 (1M) | 4 | 8.0925 | .81977 | .40989 | 6.7881 | 9.3969 | 7.09 | 9.03 |
| Total | 12 | 5.6958 | 2.01337 | .58121 | 4.4166 | 6.9751 | 3.48 | 9.03 |

| **Test of Homogeneity of Variances** | | | |  |  |
| --- | --- | --- | --- | --- | --- |
| MDA |  |  |  |  |  |
| Levene Statistic | df1 | df2 | Sig. |  |  |
| 2.429 | 2 | 9 | .143 |  |  |
| **ANOVA** | | | | | |
| MDA |  |  |  |  |  |
|  | Sum of Squares | df | Mean Square | F | Sig. |
| Between Groups | 40.312 | 2 | 20.156 | 42.404 | .000 |
| Within Groups | 4.278 | 9 | .475 |  |  |
| Total | 44.590 | 11 |  |  |  |

| **Multiple Comparisons** | | | | | | | |
| --- | --- | --- | --- | --- | --- | --- | --- |
| Dependent Variable: | MDA |  |  |  |  |  |  |
| (I) ID | | | Mean Difference (I-J) | Std. Error | Sig. | 95% Confidence Interval | |
|  |  |  |  |  |  | Lower Bound | Upper Bound |
| Tukey HSD | control | salt1 | -1.71000^*^ | .48751 | .016 | -3.0711 | -.3489 |
|  |  | salt2 | -4.45000^*^ | .48751 | .000 | -5.8111 | -3.0889 |
|  | salt1 | control | 1.71000^*^ | .48751 | .016 | .3489 | 3.0711 |
|  |  | salt2 | -2.74000^*^ | .48751 | .001 | -4.1011 | -1.3789 |
|  | salt2 | control | 4.45000^*^ | .48751 | .000 | 3.0889 | 5.8111 |
|  |  | salt1 | 2.74000^*^ | .48751 | .001 | 1.3789 | 4.1011 |
| LSD | control | salt1 | -1.71000^*^ | .48751 | .007 | -2.8128 | -.6072 |
|  |  | salt2 | -4.45000^*^ | .48751 | .000 | -5.5528 | -3.3472 |
|  | salt1 | control | 1.71000^*^ | .48751 | .007 | .6072 | 2.8128 |
|  |  | salt2 | -2.74000^*^ | .48751 | .000 | -3.8428 | -1.6372 |
|  | salt2 | control | 4.45000^*^ | .48751 | .000 | 3.3472 | 5.5528 |
|  |  | salt1 | 2.74000^*^ | .48751 | .000 | 1.6372 | 3.8428 |
| Bonferroni | control | salt1 | -1.71000^*^ | .48751 | .020 | -3.1400 | -.2800 |
|  |  | salt2 | -4.45000^*^ | .48751 | .000 | -5.8800 | -3.0200 |
|  | salt1 | control | 1.71000^*^ | .48751 | .020 | .2800 | 3.1400 |
|  |  | salt2 | -2.74000^*^ | .48751 | .001 | -4.1700 | -1.3100 |
|  | salt2 | control | 4.45000^*^ | .48751 | .000 | 3.0200 | 5.8800 |
|  |  | salt1 | 2.74000^*^ | .48751 | .001 | 1.3100 | 4.1700 |
| *. The mean difference is significant at the 0.05 level. | | | | | | | |

**MDA standard curve**

**Table S10. Statistical analysis of gene expression profile by RT-qPCR analysis in *P. indica* treated with 0.5M NaCl for 14 days.**

**Linear Regression analysis:**

| **Descriptive Statistics** | | | |
| --- | --- | --- | --- |
|  | Mean | Std. Deviation | N |
| VAR00001 | .6405 | 1.32905 | 13 |
| VAR00002 | .8154 | 2.87977 | 13 |
| **Correlations** | | | |
|  | | VAR00001 | VAR00002 |
| Pearson Correlation | VAR00001 | 1.000 | .845 |
|  | VAR00002 | .845 | 1.000 |
| Sig. (1-tailed) | VAR00001 |  | .000 |
|  | VAR00002 | .000 |  |
| N | VAR00001 | 13 | 13 |
|  | VAR00002 | 13 | 13 |

| **Model Summary^b^** | | | | | | | | | | | | | | | | |  |
| --- | --- | --- | --- | --- | --- | --- | --- | --- | --- | --- | --- | --- | --- | --- | --- | --- | --- |
| Model | R | R Square | | Adjusted R Square | | Std. Error of the Estimate | Change Statistics | | | | | | | | | |  |
|  |  |  |  |  |  |  | R Square Change | | F Change | | df1 | | df2 | | Sig. F Change | |  |
| 1 | .845^a^ | .714 | | .688 | | .74207 | .714 | | 27.492 | | 1 | | 11 | | .000 | |  |
| a. Predictors: (Constant), VAR00002 | | | | | | | | | | | | | | | | |  |
| b. Dependent Variable: VAR00001 | | | | | | | | | | | | | | | | |  |
|  |  |  | |  | |  |  | |  | |  | |  | |  | |  |
| **ANOVA^a^** | | | | | | | | | | |  | |  | |  | |  |
| Model | | Sum of Squares | | df | | Mean Square | F | | Sig. | |  | |  | |  | |  |
| 1 | Regression | 15.139 | | 1 | | 15.139 | 27.492 | | .000^b^ | |  | |  | |  | |  |
|  | Residual | 6.057 | | 11 | | .551 |  | |  | |  | |  | |  | |  |
|  | Total | 21.196 | | 12 | |  |  | |  | |  | |  | |  | |  |
| a. Dependent Variable: VAR00001 | | | | | | | | | | |  | |  | |  | |  |
| b. Predictors: (Constant), VAR00002 | | | | | | | | | | |  | |  | |  | |  |
|  |  |  | |  | |  |  | |  | |  | |  | |  | |  |
| **Coefficients^a^** | | | | | | | | | | | | | | | | | |
| Model | | | Unstandardized Coefficients | | Standardized Coefficients | | t | Sig. | | 95.0% Confidence Interval for B | | | | Collinearity Statistics | | | |
|  |  |  | B | Std. Error | Beta | |  |  |  | Lower Bound | | Upper Bound | | Tolerance | | VIF | |
| 1 | (Constant) | | .322 | .215 |  | | 1.503 | .161 | | -.150 | | .795 | |  | |  | |
|  | VAR00002 | | .390 | .074 | .845 | | 5.243 | .000 | | .226 | | .554 | | 1.000 | | 1.000 | |
| a. Dependent Variable: VAR00001 | | | | | | | | | | | | | | | | | |
|  |  |  | |  | |  |  | |  | |  | |  | |  | |  |
| **Coefficient Correlations^a^** | | | | | |  |  | |  | |  | |  | |  | |  |
| Model | | | | VAR00002 | |  |  | |  | |  | |  | |  | |  |
| 1 | Correlations | VAR00002 | | 1.000 | |  |  | |  | |  | |  | |  | |  |
|  | Covariances | VAR00002 | | .006 | |  |  | |  | |  | |  | |  | |  |
| a. Dependent Variable: VAR00001 | | | | | |  |  | |  | |  | |  | |  | |  |


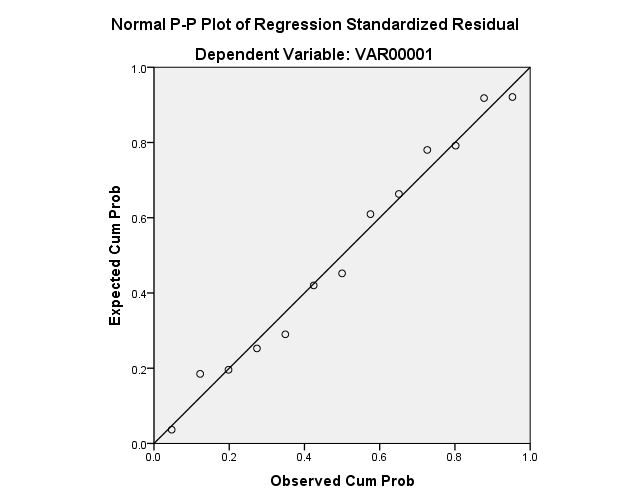

Supplement: Supplementary file 9 — Supplementary Tables. [file 41598_2021_82136_MOESM9_ESM.docx]
