# Supplementary material for: A high-throughput RNA-Seq approach to elucidate the transcriptional response of Piriformospora indica to high salt stress
Source: Sci Rep. 2021 Feb 18;11:4129. doi: 10.1038/s41598-021-82136-0 (PMC7893156; doi:10.1038/s41598-021-82136-0)
Supplement: Supplementary file 10 — Supplementary Legends. [file 41598_2021_82136_MOESM10_ESM.docx]

**Supplementary excel file:** File S (xslx)

**Supplementary Figures:**

Figure S1: Measurement of MDA content in *P. indica* treated with 0M (control), 0.5M and, 1M NaCl concentration for 7 days. Values represent the means from four replicates with bars representing ± SD. Asterisks show significance (p < 0.05) by ANOVA.

Figure S2: Significant GO terms associated with DEGs. GO classifications of overall unigenes. Most consensus sequences were grouped into three major functional categories, namely (a) biological process, (b) cellular component, and (c) molecular function.

Figure S3: Distribution of DEGs in various KOG groups. DEGs were assigned to KOG information categorized into 5 main KOG groups among which 123 unigenes belonged to metabolism, 78 unigenes to cellular processes and signaling, 58 unigenes to information storage and processing, 69 unigenes to poorly characterized and 1 unigene belonged to an undefined group.

Figure S4. GO classifications of DEGs compared to GO terms of the whole reference predicted transcriptome (green bar). GO terms with p-value <0.01 and FDR <0.05 were considered as significant.

**Supplementary Tables:**

Table S1. Dry weight of *P. indica* treated with different salt (NaCl) concentrations at different time periods.

Table S2. Measurement of MDA content in *P. indica* treated with different salt (NaCl) concentrations.

Table S3. EuKaryotic Orthologous Groups (KOG) class enrichment for DEGs in *P. indica* after treatment with 0.5M NaCl for 14 days.

Table S4. List of DEGs related transporter proteins and transcription factors in *P. indica* treated with 0.5M NaCl for14 days.

Table S5. List of salt-responsive differentially expressed genes in *P. indica* after treatment with 0.5M NaCl for 14 days.

Table S6. Unigenes used for validation of gene expression profile by RT-qPCR analysis in *P. indica* transcriptome data.

Table S7. Primers used in RT-qPCR analysis for validation of gene expression profile in *P. indica* transcriptome data.

**Additional supplementary information:**

Table S8. Statistical analysis of *P. indica* dry weight measurements treated with different salt (NaCl) concentrations over different time intervals.

Table S9. Statistical analysis of MDA content measurements in *P. indica* treated with different salt (NaCl) concentrations.

Table S10. Statistical analysis of gene expression profile by RT-qPCR analysis in *P. indica* treated with 0.5M NaCl for 14 days.
